# A spatiotemporal atlas of organogenesis in the development of orchid flowers

**DOI:** 10.1093/nar/gkac773

**Published:** 2022-09-12

**Authors:** Chang Liu, Jing Leng, Yonglong Li, Tingting Ge, Jinglong Li, Yamao Chen, Chunce Guo, Ji Qi

**Affiliations:** State Key Laboratory of Genetic Engineering, School of Life Sciences, Fudan University, Shanghai, China; State Key Laboratory of Genetic Engineering, School of Life Sciences, Fudan University, Shanghai, China; Jiangxi Provincial Key Laboratory for Bamboo Germplasm Resources and Utilization, Forestry College, Jiangxi Agricultural University, Nanchang, China; Jiangxi Provincial Key Laboratory for Bamboo Germplasm Resources and Utilization, Forestry College, Jiangxi Agricultural University, Nanchang, China; State Key Laboratory of Genetic Engineering, School of Life Sciences, Fudan University, Shanghai, China; State Key Laboratory of Genetic Engineering, School of Life Sciences, Fudan University, Shanghai, China; Jiangxi Provincial Key Laboratory for Bamboo Germplasm Resources and Utilization, Forestry College, Jiangxi Agricultural University, Nanchang, China; State Key Laboratory of Genetic Engineering, School of Life Sciences, Fudan University, Shanghai, China

## Abstract

Development of floral organs exhibits complex molecular mechanisms involving the co-regulation of many genes specialized and precisely functioning in various tissues and developing stages. Advance in spatial transcriptome technologies allows for quantitative measurement of spatially localized gene abundance making it possible to bridge complex scenario of flower organogenesis with genome-wide molecular phenotypes. Here, we apply the 10× Visium technology in the study of the formation of floral organs through development in an orchid plant, *Phalaenopsis* Big Chili. Cell-types of early floral development including inflorescence meristems, primordia of floral organs and identity determined tissues, are recognized based on spatial expression distribution of thousands of genes in high resolution. In addition, meristematic cells on the basal position of floral organs are found to continuously function in multiple developmental stages after organ initiation. Particularly, the development of anther, which primordium starts from a single spot to multiple differentiated cell-types in later stages including pollinium and other vegetative tissues, is revealed by well-known MADS-box genes and many other downstream regulators. The spatial transcriptome analyses provide comprehensive information of gene activity for understanding the molecular architecture of flower organogenesis and for future genomic and genetic studies of specific cell-types.

## INTRODUCTION

Development of floral organs is a mysterious process unique in angiosperms, involving a series of cell activities like cell growth, division and differentiation at specific locus as well as cell–cell interactions (1,2). Despite of the species-specific matters, the successive dynamic process of organogenesis of the four whorls: sepal, petal, stamen and carpel, is usually segmented into four stages: initiation of floral primordium from inflorescence meristems, identity determination of organs, morphogenesis based on cell expansion and differentiation, and maturation (2). After decades of efforts on studying floral development in *Arabidopsis thaliana* (3–5), *Antirrhinum majus* (6), *Oryza sativa* (7) and other few plants, hundreds of genes have been identified to play essential roles in the formation of flowers, including genes coding for phytohormones, transcription factors and epigenetic regulators (8,9). Among them, MADS-box genes, well known for transcription regulations in normal development and cell differentiation in animals, e.g. for development of gastrula (10), muscle cell (11), neural system and varying tissues (12), are also thoroughly explored in plant studies. Their functions in plants are recognized for the determination of four whorls of floral organs summarized as ‘ABC’ model (13) and later extended ‘ABCDEG’ model (14). The ‘ABC’ model follows a rule, that is, the formation of the primordia of sepal, petal, stamen and carpel is determined by the expression of A-function, A + B, B + C and C-function genes, respectively. Recent studies have set up a system-wide gene regulatory network for *Arabidopsis thaliana* flower development (15). However, the molecular mechanisms underlying floral development among diverse angiosperms varying in morphologies, development, function and pollination and fertilization are still unknown, not to mention that even closely related species may exhibit different morphological characters of the same organs to adapt to adequate pollinators as a result of co-evolution.

The orchid family (Orchidaceae) is one of the largest families of angiosperms, with 763 genera and over 28 000 species, distributing worldwide. These species account for ∼10% of all flowering plants with diverse habitats and floral structures, making the orchid family considered as a model family for studying the development and evolution of floral organs. A typical orchid flower is composed of three types of perianth organs: three outer tepals, two inner tepals, and a labellum (or lip). A hypothesis of orchid floral development assumes that the identities of various perianth organs are determined by the combinatorial interaction of four AP3-like genes (16), yielded from two rounds of gene duplication events during the early evolution of orchids (17). Nevertheless, comprehensive understanding of the complex process of orchid floral development is still far from complete, especially lacking a spatiotemporal survey of key regulatory networks for organogenesis.

Recent advances in spatial transcriptome technologies, e.g. *in situ* hybridization based MERFISH (18) and in situ sequencing based 10× Visium and STARmap (19), have opened a new era in the field of genetics and genomics, by providing a high-resolution map of gene expression pattern in each bead/spot. Here we present a study which exhibits spatiotemporal gene expression profiles among multiple developmental stages of *Phalaenopsis* Big Chili flowers by using 10× Visium technology. Detailed examination of thousands of genes with distinct expression strength or preferential patterns on different stages, provides a spatiotemporal atlas of the initiation of floral meristems and early development of floral organs with conserved characteristics across species, and also tissues specific in orchids, e.g. lips and columns. The high resolution spatial transcriptomic data also reveals the successive activity of floral meristems during the development of different organs, and cell differentiation from meristematic cells to vegetative or reproductive cells. These results as well as thousands of marker genes spanning many tissues in multiple development stages, provide valuable resources for further genetic and genomic investigation of the complex scenario in flower development.

## MATERIALS AND METHODS

### Plant tissue dissection and spatial transcriptome sequencing on 10× Visium platform

Inflorescences of *P*. Big Chili spanning multiple floral stages were collected from plants from the plant facility of School of Life Sciences, Fudan University. The samples were prepared, embedded and cryosectioned according to the following steps (20):

1. Immerse the fresh buds in 1ml EAA (ethanol (EtOH > 99%):acetic acid = 3:1) and vacuum on ice for 10 min (at 10–100 mbar).
2. Transfer the tissues into 1 ml 10% sucrose solution with 10× PBS buffer (80 g NaCl, 14.7 g Na_2_HPO_4_·12H_2_O, 2 g KCl, 2 g KH_2_PO_4_, to 1 l with RNase free water, pH to 7.4), vacuum on ice for 20 min, and shake (20 rpm) the sample at 4°C until the tissues sink (40–60 min).
3. Replace the solution with 20% sucrose solution and repeat step 2.
4. At room temperature, fill the embedding moulds with optimal cutting temperature (OCT) compound (Sakura finetek Europe B.V.).
5. Gently dry the solution on the tissues’ surface, and soak the tissues in OCT. Wait 15 min so that the residual sucrose solution on the tissues’ surface is replaced with OCT.
6. Adjust the position of the tissues in OCT to ensure an appropriate section plane.
7. Immerse the bottom of the embedding mould in isopentane pre-cooled with liquid nitrogen.
8. Take out the mould when OCT is fully frozen and store the sample in a −80°Cfreezer.
9. Slice the sample at −20°C cryochamber, the section thickness of 20 μm is adopted in this study.

Tissue sectioning, Trypan staining, tissue permeabilization, fluorescent cDNA synthesis and imaging were performed on 10× Visium platform with resolution of 55 μm, according to the manufacturer's protocol. cDNAs of samples were sequenced on Illumina novaseq platform and were subjected to 150 cycles of paired-end (2 × 150 bp) sequencing. The first sample includes the shoot apical meristem and 6 terminal buds. As the buds in the first sample show successive development stages, following the definition of *A. thaliana* (3) floral organogenesis and the morphological characteristics, we simply name them as: bud 1, flower meristem arises; bud 2, flower primordium forms; bud 3, outer tepal primordia arise; bud 4, outer tepal overlies flower meristem; bud 5, column primordia arise; bud 6, pollinium arises. The second and the third samples for dissection include single buds, defined as bud 7 (lip projection and column foot form) and bud 8 (floral organs become mature) in this study, were collected from independent plant individuals.

The *Phalaenopsis aphrodite* reference genome and corresponding gene annotation models were downloaded from *P. aphrodite* Genome Resources (21) for the mapping of NGS short reads and measurement of gene expression. Sequenced reads were trimmed by SpaceRanger (https://support.10xgenomics.com/spatial-gene-expression/software/overview/welcome) of 10× Genomics and mapped to the reference genome using STAR with default settings(22). Unique molecular identifiers (UMIs) for each spot were counted to remove PCR duplicates. Genome divergence between *P*. Big Chili and *P. aphrodite* was estimated from the mapping results of UMIs of all samples using GATK (23) with a cutoff of read depth as 5.

### Identification of cell-types from spatial transcriptomic datasets

We employed STEEL (https://doi.org/10.21203/rs.3.rs-1240258/v1) for recognition of cell-types/domains from the spatial transcriptomes. STEEL adopts a five-step strategy for clustering: (i) identifies spatially varying genes measured by spatial Gini coefficient score; (ii) calculates the conditional probability matrix *P* to represent similarities of paired beads in high-dimensional expression space; (ii) calculates the conditional probability matrix *Q* denoting similarities of paired beads in two-dimensional space of the histological section in a manner of graph; (iv) calculates the cross-entropy to measure the faithfulness of modeling *P* with *Q*, and the adjacent paired beads/subgraphs with minimal increase of entropy is merged into one larger group; (v) performs clustering step iteratively until all adjacent subgraphs are merged.

STEEL was applied on all three samples with default parameters: bead occupancy of genes higher than 0.05%, gene occupancy of beads higher than 0.5%, number of neighbors as 20; perplexity as 35. To reduce the noise on detected gene expression, an option ‘–pca = 20’ was applied to employ principal component analysis on the expression of spatially varying genes with Gini score higher than or equal to 0.5. The clustering of spots/beads for each slide was based on the PCA transformed matrix. Cell-types were annotated based on both expression of known marker genes and histological information on Trypan blue stain sections. At last, a 40-group classification was adopted for each dataset, to present a clear segmentation of different tissues and to avoid yielding groups with scattered beads caused by noises on expression signals.

### Hierarchical clustering of global gene expression profiles and PCA analysis

After the clustering procedure, an average expression of each gene across spots of the same cluster was counted. A global gene expression profile was then built for each cluster based on all spatial varying genes on a given dataset. Pearson's coefficient was adopted as a measure of similarity for each pair of clusters. Hierarchical relationship of all clusters was estimated by using hclust package in R with clustering method of ‘complete’. To facilitate comparisons among clusters from different sections, all spatially varying genes were combined together for building gene expression profiles. The merged expression matrix was scaled and centered for PCA analyses using prcomp package in R.

### Track of cell development trajectory on sub-datasets of specific clusters

To discover cell development trajectory of floral tissues of early stage, spots of clusters 19, 21, 37, 38, 39 and 40 were isolated from original dataset of slide 1 by using custom scripts. The data subset includes floral meristem cells and also primordium of tepal, lip and column. Trajectory analysis was performed by using Monocle (24) with options as: ‘min_expr = 0.1’ for function ‘detectGenes’, ‘mean_expression ≥ 0.1’ for function ‘subset’, ‘max_components = 2, reduction_method = ‘DDRTree’’ for function ‘reduceDimension’. Pseudotime related genes were obtained by function ‘differentialGeneTest’ with option ‘cores = 5’ and ‘qval < 0.01’. Heatmap analysis was performed by using function ‘plot_genes_branched_heatmap’ with options ‘num_clusters = 6, cores = 1’ All the other parameters were set as default.

For trajectory analysis of floral meristem and tepal tissues, spots of clusters 1, 2, 22 and 27 were isolated from slide 1 as input dataset of Monocle with parameters as described above. A heatmap was obtained by using function ‘plot_pseudotime_heatmap’ with option ‘num_clusters = 4’.

### Clustering of genes co-expressed in anther tissues

For each sample, STEEL output a gene expression profile for each cluster by averaging the expression values of all beads/spots in the cluster. A list of 1683 genes related to anther development was collected, including those preferentially expressed in cluster 36 of sample 1 (anther of bud 6), cluster 21 in sample 2 (pollinium of bud 7) and cluster 28 in sample 3 (pollinium of bud 8). As the cluster 38 of sample 1 (column of bud 5) was composed of a single bead denoting for anther primordium, the expression values of the 1683 genes in the cluster were also involved for comparison. The normalized expression of the 1683 genes on the four tissues was further segmented into six groups by using K-means methods in R.

### Functional enrichment analysis

MapMan functional categories (25) of all protein coding genes of the *P. aphrodite* genome were obtained using Mercator4 (26). Gene Ontology (GO) terms of these genes were annotated by eggNOG-mapper (27) with default parameters. MapMan functional category and GO term enrichment analysis for clustered genes was examined by comparing with total genes on the genome using clusterProfiler 4.0 (28) based on Fisher's exact test with the false discovery rate (FDR) < 0.05.

### Ortholog identification and reconstruction of phylogenetic tree for selected gene families

To obtain detailed phylogenetic relationship of type II MADS-box genes of *P. aphrodite*, all-against-all blastp was performed for protein sequences of seven angiosperm species from the Phytozome database (29): *Phalaenopsis aphrodite*, *Phalaenopsis equestris*, *Apostasia shenzhenica*, *Cymbidium ensifolium*, *Oryza sativa*, *Arabidopsis thaliana* and *Amborella trichopoda*. Hierarchical ortholog groups were then predicted by using PhyloMCL (30) with default parameters. Annotated type II MADS-box genes from the seven species were adopted for recognizing related gene families. Multiple sequence alignment was performed for proteins of MADS-box genes using MUSCLE v3.8.31 (31) with default parameters. Aligned matrix was trimmed by trimAI v1.4 (32) with options ‘-gt 0.1 -resoverlap 0.75 -seqoverlap 80’ and was transferred to a nucleic acid alignment matrix by PAL2NAL (33). A maximum-likelihood tree was reconstructed using RAxML (34) with evolutionary model of ‘‘GTRCAT,’’ and a bootstrap significance test was performed with 100 replicates.

As for large gene families, i.e. for auxin response factor (ARF), AUX/IAA, auxin inducible protein, cytokinin oxidase, cytokinin-responsive gata factor, bHLH and MYB, only four species were employed for reconstruction of phylogenetic trees: *P. aphrodite*, *O. sativa*, *A. thaliana* and *A. trichopoda*. Procedures of homolog clustering, multiple sequence alignment, matrix refinement and gene tree reconstruction are as same as above.

### Transcription comparison between spatial transcriptome and bulk RNA-seq technologies

To compare gene expression levels of orchid buds inferred from bulk RNA-seq and spatial transcriptomes in this study, a dataset of small flower bud of *P. aphrodite* was downloaded from *P. aphrodite* Genome Resources (21) (NCBI accession: SRR4302012). The sequenced reads were mapped against the *P. aphrodite* genome by HISAT2 (35), and gene expression levels were estimated using StringTie (36). FPKM values of the 12,586 genes detected both on the bulk RNA-seq data and on the slide 3 of this study were adopted for comparison of expression values.

## RESULTS

### Spatial transcriptome sequencing of the early development of orchid floral organs and cell-type recognition

**Figure 1.**
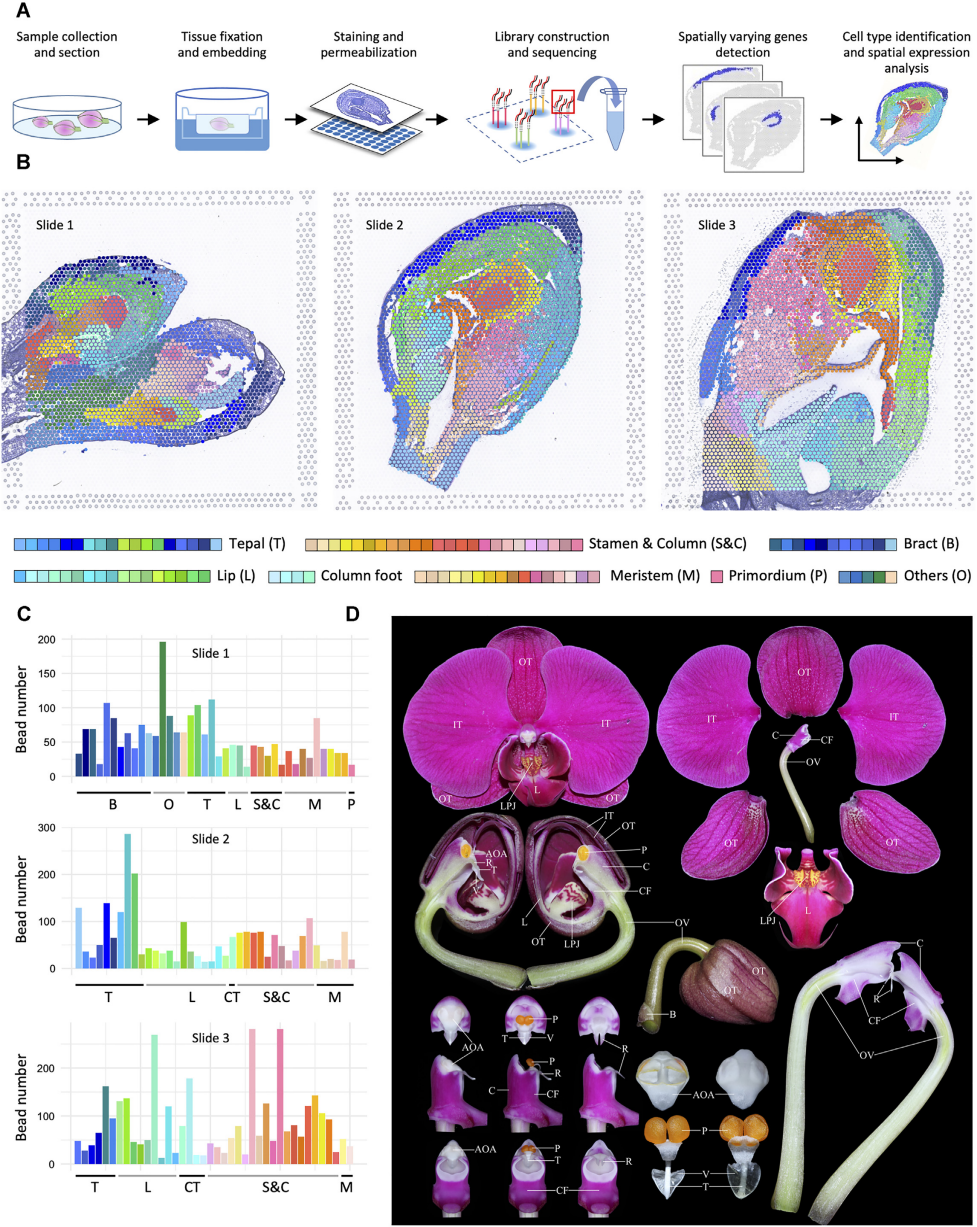
Reconstruction of organogenesis in early developmental stages of orchid flower based on spatial transcriptomics. (**A**) A workflow for sampling and sequencing of *Phalaenopsis* Big Chili flower on a 10X Visium platform. (**B**) Illustration of discovered cell-types on the three slides, placing over corresponding Trypan blue stain images. Positions of buds in the first slide are shown in Supplementary Figure S1. (**C**) Histogram for bead numbers of each cell-type discovered in (B) with consistent colors. (**D**) Illustration of different organs of *P*. Big Chili flowers. AOA, apex of anther; B, bract; C, column; CF, column foot; FM, floral meristem; FP, floral primordium; IM, inflorescence meristem; IT, inner tepal; L, lip; LP, lip primordium; LPJ, lip projection; OT, outer tepal; OTP, outer tepal primordium; P, pollinium; R, rostellum; T, tegula; V, viscidium.

Floral organogenesis starts from the formation of floral meristem, followed by the subsequent initialization, identity determination and morphogenesis of the organs of the four whorls until the maturation (37). To provide a spatiotemporal cell atlas of flower organogenesis for comprehensive understanding of the complicated scenario, we performed spatial transcriptome sequencing on the sagittal planes of three samples of the racemose inflorescence tissues of *P*. Big Chili, spanning most of the early developmental stages from the existence of inflorescence meristem to nearly matured floral organs on a 10× Visium platform (Figure 1A). Among the 194–199 million reads for each sample, 85.7–86.9% of them were mapped onto the *P. aphrodite* reference genome (Supplementary Table S1), yielding 325 020 SNPs in the genic regions, with 185 907 SNPs (57.2% of the total) in exonic regions and the other 42.8% in intronic regions.

**Figure 2.**
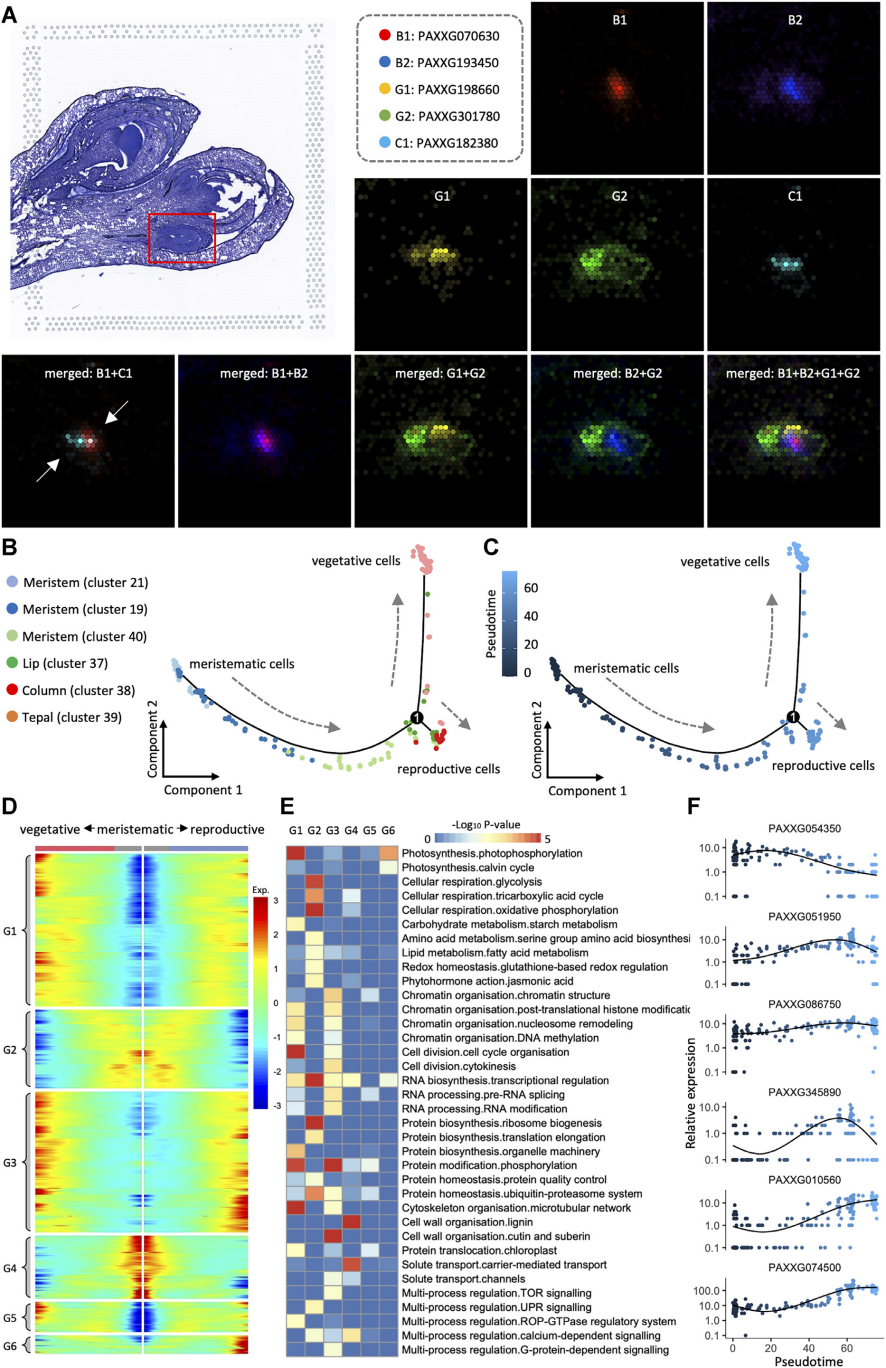
Differentiation from meristematic cells to vegetative and reproductive cells. (**A**) Spatial expression of five MADS-box genes on flower bud 5 of slide 1 (Supplementary Figure S1). (**B** and **C**) Illustration of pseudotime trajectory of cell-type profiles for six clusters of flower bud 5. (**D**) Heatmap of 4685 genes differentially expressed before and after the branch point in (B). (**E**) MapMan functional enrichment for different clusters of genes involved in cell fate determination. (**F**) Illustration of six genes correlated with estimated pseudotime.

The first sample (Supplementary Figure S1) exhibits the earliest stages of floral development, consisting of tissues of inflorescence meristem and six buds of successive development stages from floral meristem, distinguishable primordia of tepal, column and lip to the developing organs of anther and accessory structure of lips. Spatial transcriptome sequencing of the sample results in 2233 valid beads (owning at least 0.5% of all expressed genes) and 13,311 valid genes, among which 3,406 genes (Supplementary Table S2) exhibit significant spatial variation among different tissues (left panel of Figure 1B). Application of STEEL on the data clearly classifies these beads into 40 clusters of tepals (six clusters), lips (three clusters), stamens and columns (five clusters), meristems and primordia (10 clusters), bracts (11 clusters) and other tissues (five clusters). Detailed examination of the spatial expression patterns of known genes, specifically or preferentially expressed in one or more tissues, well supports the classification, e.g. the groups denoting for tepal (cluster 39, Supplementary Figures S2 and S3), lip (cluster 37) and meristem (cluster 40) exhibit high consistency with the spatial expression of PAXXG301780 and PAXXG198660 (AGL6-like) of *P*. Big Chili (Figure 2A). Similarly, the expressions of PAXXG070630 (AP3-like), PAXXG193450 (PI-like) and PAXXG182380 (AG-like) well support the cluster for stamen and column (cluster 38, Supplementary Figure S2).

The second sample covers an entire bud at a relatively later development stage (Supplementary Figure S4), owning 2461 valid beads and 13 071 valid genes, of which 1116 ones show spatial variation on expression (Supplementary Table S3). A 40-group clustering of the plane (middle panel of Figure 1B, Supplementary Figures S5 and S6) yields cell-types representing for relatively matured floral organs including tepals (10 clusters), lip (12 clusters), stamen and column (11 clusters), column foot (1 cluster) and meristems (6 clusters). Beads from tissues of tepal, lip and column are well clustered based on global expression similarities of spatially varying genes, and are highly consistent with observations on the corresponding histological examination (Supplementary Figure S4). Furthermore, pollinium is undergoing process of morphogenesis, during which preferential expressions of over 400 genes, potentially involved in normal development of anther, are identified, including PAXXG086750 (homologous to *AtAGO4* (38)) and PAXXG129450 (homologous to *AtMBF1C* coding for protein bridging factor in hormone metabolism (39)). Many differentiated tissues, e.g. rostellum and lip projection (accessory of lip), are emerged with considerable sizes and shapes, outlined by hundreds of preferentially expressed genes which are potentially responsible for the initialization and morphogenesis of these organs.

The third slide is composed of a bud of late morphogenesis stage with most of its organs nearly formed (Supplementary Figure S7). The outer tepals were removed before tissue fixation and embedding to fit in the slide. The sequenced tissues cover 3394 beads with 13 091 expressed genes (1816 of them with Gini score ≥0.5, Supplementary Table S4). Highly consistent with histological observations, stamen and column tissues own the most diversity occupying 19 of the total 40 clusters (right panel of Figure 2B, Supplementary Figures S8 and S9), far more than those relatively matured organs like inner tepals and lip with six and nine clusters, respectively.

In summary, the spatial transcriptomes of the three slides detect the expression of 14 328 genes, among them 3817 genes are identified as specifically or preferentially expressed in one or more tissues. These sections cover a continuous developing process from the initialization, identity determination to late morphogenesis stages of orchid flowers, providing a molecular atlas for subsequent detailed analyses of cell-type changes from meristematic cells to differentiated ones.

### Presence of meristematic cells on basal position of early buds and their subsequent differentiation into vegetative and reproductive cells

As shown in Supplemental Figure S1, tissues of inflorescence and floral meristems and the adjacent bracts are recognized according to the morphological observation. However, these meristematic cells of the earliest budding stages show highly homogeneity on global gene expression, molecularly, they are indistinguishable and are grouped together (Figure 1B and Supplementary Figure S1) until later in the larger bud 4, the primordia of tepals and lip are then identified by different expressions of many genes, e.g. PAXXG117100, a homolog of *AtGPR7* important for floral transition and initiation of *A. thaliana* (40).

When more floral organs like tepal, lip and column emerge in the larger buds (e.g. buds 5 and 6, Supplemental Figure S1), the meristematic cells, represented by beads of clusters 21, 19 and 40 (left panel of Figures 1B, 2A and Supplemental Figure S2), continue to function on the basal position of floral organs even in the largest bud 8 when morphogenesis is nearly complete. To study the cell differentiation from meristematic cells to vegetative cells (tepals and lip) and reproductive ones (column), trajectory of cell states is referred using Monocle (24). As shown in Figure 2B, the three clusters of meristematic cells form a continuous path, representing the developing process which is highly consistent with their positions on the histological section (Supplemental Figure S1). The path then divides into two sub paths: one is composed of vegetative cells of tepals and the other is of column including reproductive cells of anther primordium recognized by multiple MADS-box genes (Figure 2A). The trajectory of cell-type changes, as a static screenshot of a dynamic developing process, can also be interpreted as a pseudotime differentiation of the meristematic cells, as shown in Figure 2C, when vegetative cells emerge earlier and scatter at the far end of the sub path. In details, a total of 4685 spatially varying genes are identified to be correlated with the pseudotime of development, and exhibit different expression patterns along with the separation of the development paths. These genes are further clustered into different groups (Figure 2D), which are enriched in MapMan function categories for metabolisms of cell wall, hormone, lipid, protein and RNA synthesis (Figure 2E) and are overrepresented in GO function categories of cell cycle and plant organ morphogenesis (Supplemental Figure S10). As for the two types of phytohormones playing essential roles in early differentiation of floral organs (1), 44 auxin related genes display expression signals preferentially either in meristematic cells or differentiated ones (Supplemental Figure S11), likewise, 12 cytokinin related genes exhibit similar preferential expression patterns, hypothetically interacting with the correlated auxins to initiate the formation or differentiation of floral organ cell.

Besides these well-known genes, many others are detected to be involved in meristematic, vegetative and reproductive tissues and might also function in the cell differentiation process (Figure 2F). In particular, a gene PAXXG054350 (homologous to *ATAUX2-11* coding for auxin inducible related transcription factor (41)) is specifically expressed in meristematic tissues, with no signal in the differentiated ones, while another two genes, PAXXG051950 (homologous to *AtANT* essential for the control of cell proliferation (42)) and a transcription factor PAXXG086750 (homologous to *AtAGO4*), are not only highly expressed in meristematic cells, but appear in vegetative ones as well. Moreover, some genes may be potentially involved in the cell differentiation from meristematic to vegetative tissues, like PAXXG345890 (homologous to *AtLFY* related to expression regulation of AP3 family genes (43)). Nonetheless, with the organs developing, more genes show increasing expression levels in differentiated cells. For example, a homeobox gene PAXXG010560 (homologous to *AtPDF2* for floral organ development and maturation (44)) is expressed in primordia of both tepal and column, and PAXXG074500 of cytochrome P450 superfamily (homologous to *AtCYP78A5*) shows higher expression in column than in tepals.

### Spatial expressions of MADS-box genes illustrating ABC codes of orchid floral organs for identity determination

**Figure 3.**
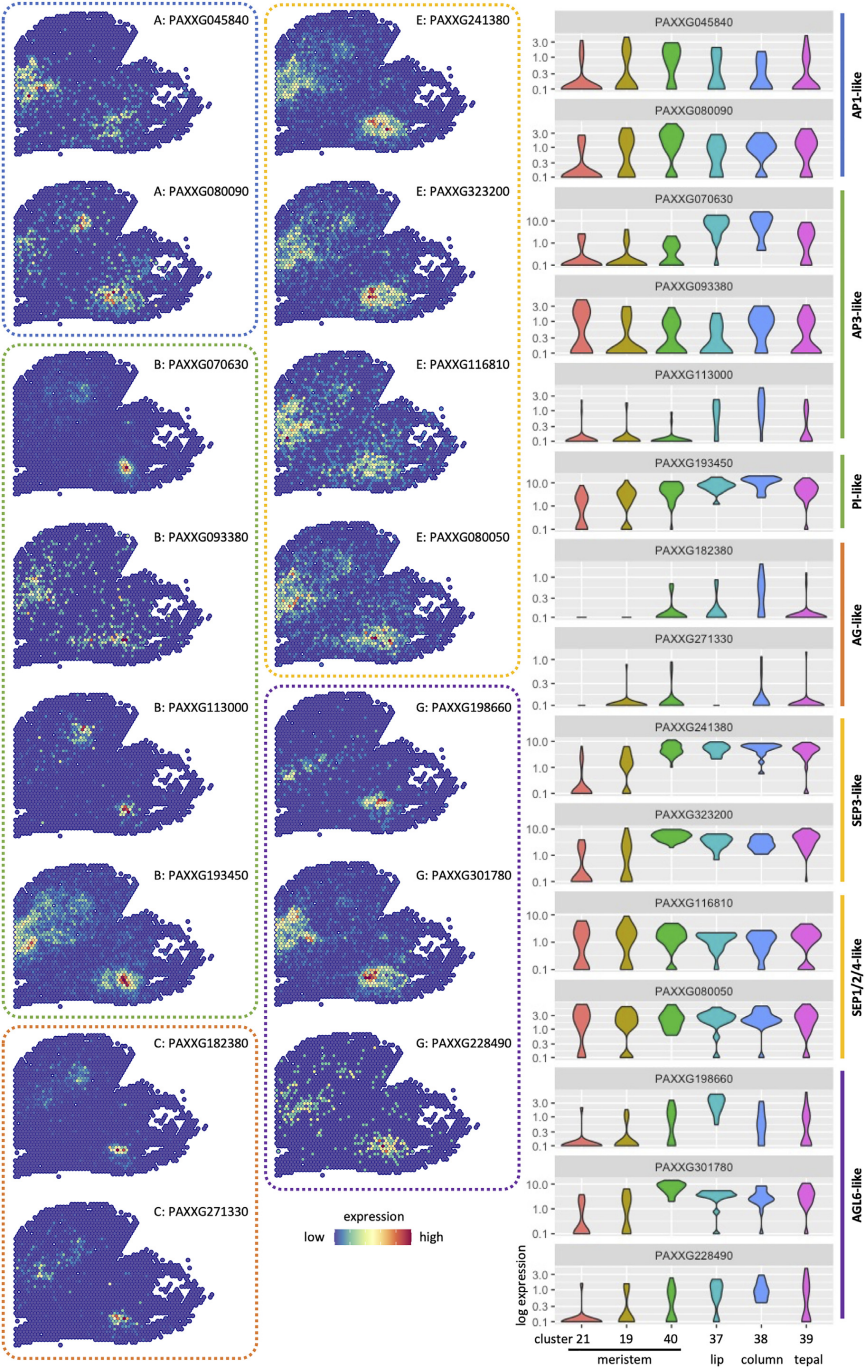
Illustrations of spatial expression distributions for 15 MADS-box genes. Quantitative expression levels of the 15 MADS-box genes on different tissues are presented on the right panel. The six clusters denoting for meristem, lip, column and tepal are consistent with those in Figure 2B.

To discover the specific ABC code in orchid of petaloid monocot, we performed the phylogenetic analysis of MADS-box genes among seven angiosperm species, including four orchids (*P. aphrodite*, *P. equestris, A. shenzhenica* and *C. ensifolium*), one grass plant (*O. sativa*), one eudicot (*A. thaliana*) and one basal angiosperm (*A. trichopoda*) (Supplemental Figure S12). Among all 21 ABCDEG class MADS-box genes recognized in *P*. Big Chili, 15 of them have been detected with expression signals on the three spatial transcriptomic datasets (Figure 3).

Functions of B- and C-class genes, essential for both vegetative and reproductive identities, are highly conserved among angiosperms (45). Previous studies on *P. equestris* suggest that both AP3- and PI-like genes (B-class) are comprehensively expressed in primordia of tepals, lip and column (46). Consistently, the three AP3-like genes of *P*. Big Chili present preferential and different expression patterns revealed by spatial transcriptomic data. Among them, the expression of PAXXG070630 and PAXXG113000 is higher in column than in lip and rare in tepal, similar to the expression pattern of its homolog in *P. equestris*, *PeMADS3*, which is higher in lip than in column and shows no signals in tepal (47). Also for PAXXG093380, consistent with its homolog *PeMADS5* in *P. equestris* (tepal ∼ column > lip) (47), it shows higher expression in basal meristematic tissues (cluster 19 of slide 1, Figures 2A and 3), column and tepal than in lip. These results suggest that AP3-like genes of *P*. Big Chili, although owning highly similar sequences yielded from gene duplication events on the ancestor of orchid, have undergone functional divergence, leading to the spatiotemporal variation on the transcription regulation of development. In particular, PAXXG070630 exhibits gradient expression signals around the tip of column tissue where a single bead shows the highest expression values and its surrounded cells have decreased signals along with their distance to the center bead, indicating the initiation of anther from a single spot at this very moment of development stage. Furthermore, the same bead is also highlighted on the spatial expression profile of PAXXG182380 (AG-like, C-class), providing further evidence of the organogenesis of anther. Meanwhile, the other highlighted bead on the profile provides strong evidence for the development of carpal primordium (Figures 2A and 3). A PI-like gene (PAXXG193450) of the other lineage of B-class, is widely expressed in meristematic tissues and multiple initialized organs with higher expression in inner whorls than in outer whorls (Figures 2A and 3), suggesting its correlation with the normal development of both vegetative and reproductive tissues.

In *A. thaliana*, *Antirrhinum majus* and many other eudicots, the A-function, essential for identity determination of sepal and petal, is carried out by multiple duplicated A-class genes (AP1). However, AP1-like genes in *P*. Big Chili are preferentially expressed in meristem or widely expressed among various organs rather than the tepal primordium and the subsequent initialized tissues (Figure 3). Instead, in orchid, A-function for identity determination of two identical whorls homologous to petal, is performed by two AGL6-like G-class genes PAXXG198660 and PAXXG301780, which specifically express in lip and tepals, respectively (Figure 3) (48). These two genes, as a result of a duplication event before the divergence of orchid family (Supplemental Figure S12), must have undergone functional divergence for their unique roles in the origination and formation of different vegetative organs.

### Growth of tepals from continuously activated meristematic cell groups

**Figure 4.**
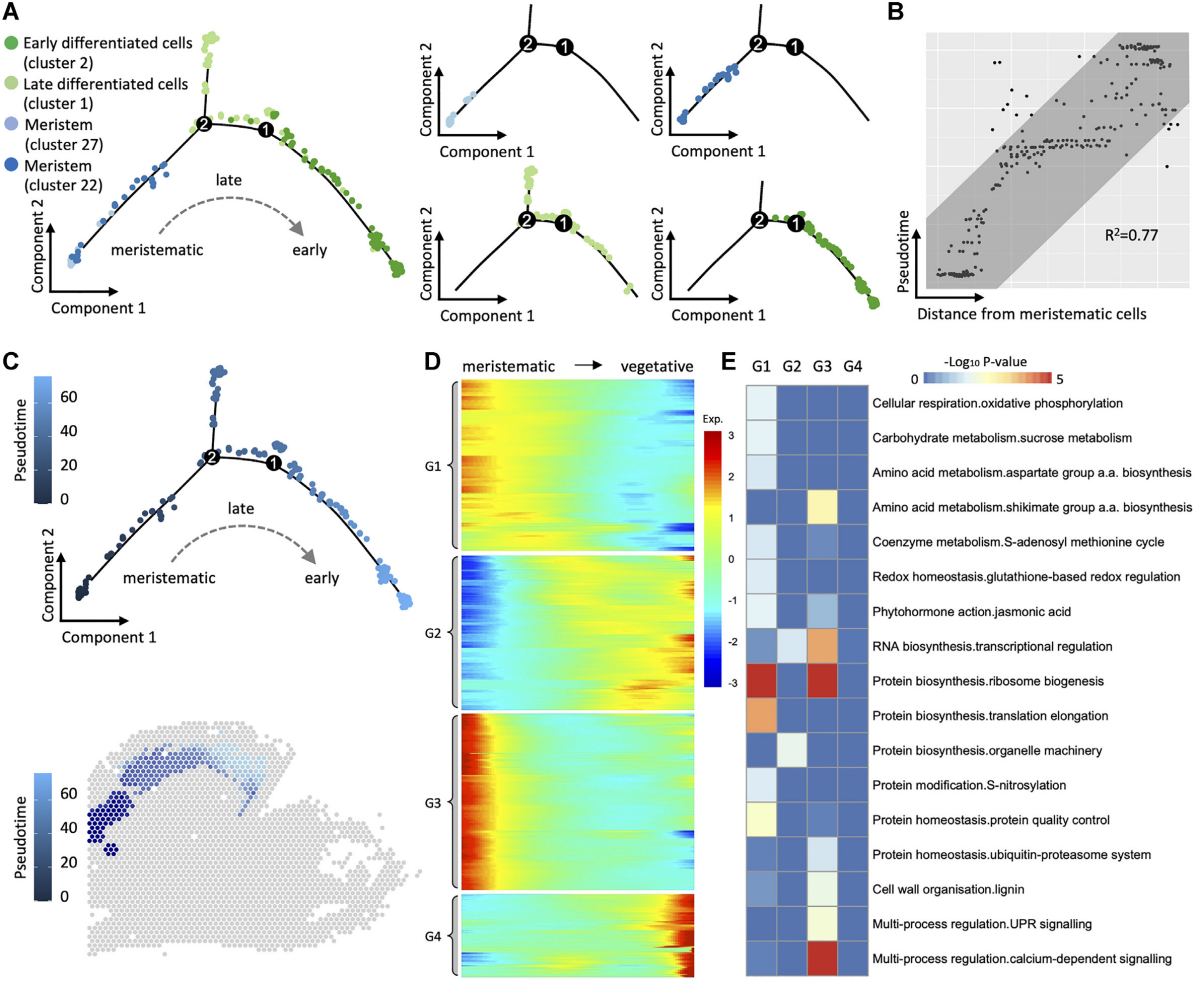
Analyses of cell differentiation from meristematic cells to tepal cells of bud 6 on slide 1. (**A**) Pseudotime estimation for four clusters of bud 6 discovered on slide 1. (**B**) Spot plot for the correlation between pseudotime and position of each bead. (**C**) Presentation of pseudotime on estimated trajectory paths for the four clusters. (**D**) Heatmap of 987 genes differentially expressed between meristematic and vegetative cells. (**E**) MapMan functional enrichment for different clusters of genes involved in cell fate determination.

After identity determination, vegetative tissues, particularly the tepals start the process of morphogenesis first and are adopted for subsequent analysis. Tepal primordium, initialized at bud 3, is elongated at bud 4, in which period, the tepals are big enough to enclose the lip primordium. However, at this stage, the tepals and the primordium of lip show high degree of homogeneity in global gene expressions, thus are grouped into one cluster, which is distinguished from surrounding tissues based on the preferential expression of 62 genes involved in DNA synthesis, transcription regulation, cell organization and signal transduction, etc. In bud 5, tepal cells are singled out from other floral organs by the strong signals of a G-function gene (PAXXG301780, Figure 2A) and are grouped at the far end of the trajectory path (Figure 2B). However, to further investigate the morphogenesis process of tepal tissues, one tepal from bud 6 is selected. The tepal tissue is much larger than those of bud 5, and consists of 4 clusters with both meristematic (clusters 22, 27) and vegetative (clusters 1, 2) cells, exhibiting high heterogeneity among cells (Supplementary Figure S2). As illustrated in Figure 4A, the trajectory analysis reveals that the development starts from the basal meristematic cells (cluster 22), followed by cells of cluster 27, then by late (cluster 1) and early (cluster 2) differentiated tepal cells scattered along the path. Estimated pseudotime analysis of these beads based on their global gene expression is highly correlated with their arc distance to the meristematic cells on the slide (Figure 4B and C), indicating a continuous change of cell status from meristem to late differentiated cells then to early differentiated cells. A list of 987 genes is discovered to be correlated with estimated pseudotime of these cells, shows either increasing expression levels along with pseudotime or decreasing ones (Figure 4D). Genes preferential in meristem are enriched in MapMan categories of protein synthesis, hormone metabolism and redox related processes (Figure 4E), and in GO terms response to stimulus (Supplemental Figure S13). On the contrary, genes preferential in tepal tissues are enriched in light reaction or mitochondrial electron transport, indicating the activities of photosynthesis on differentiated cells. The presence of meristematic cell groups on the basal position of floral organs and the heterogeneity of gene expression on early-/late- differentiated cells, e.g. PAXXG046700 (homologous to *AtFAD8* related to lipid metabolism (49)) and PAXXG237140, clearly reveal the process of early morphogenesis development of tepal tissues.

### Initiation of anther and subsequent differentiation of multiple cell-types

**Figure 5.**
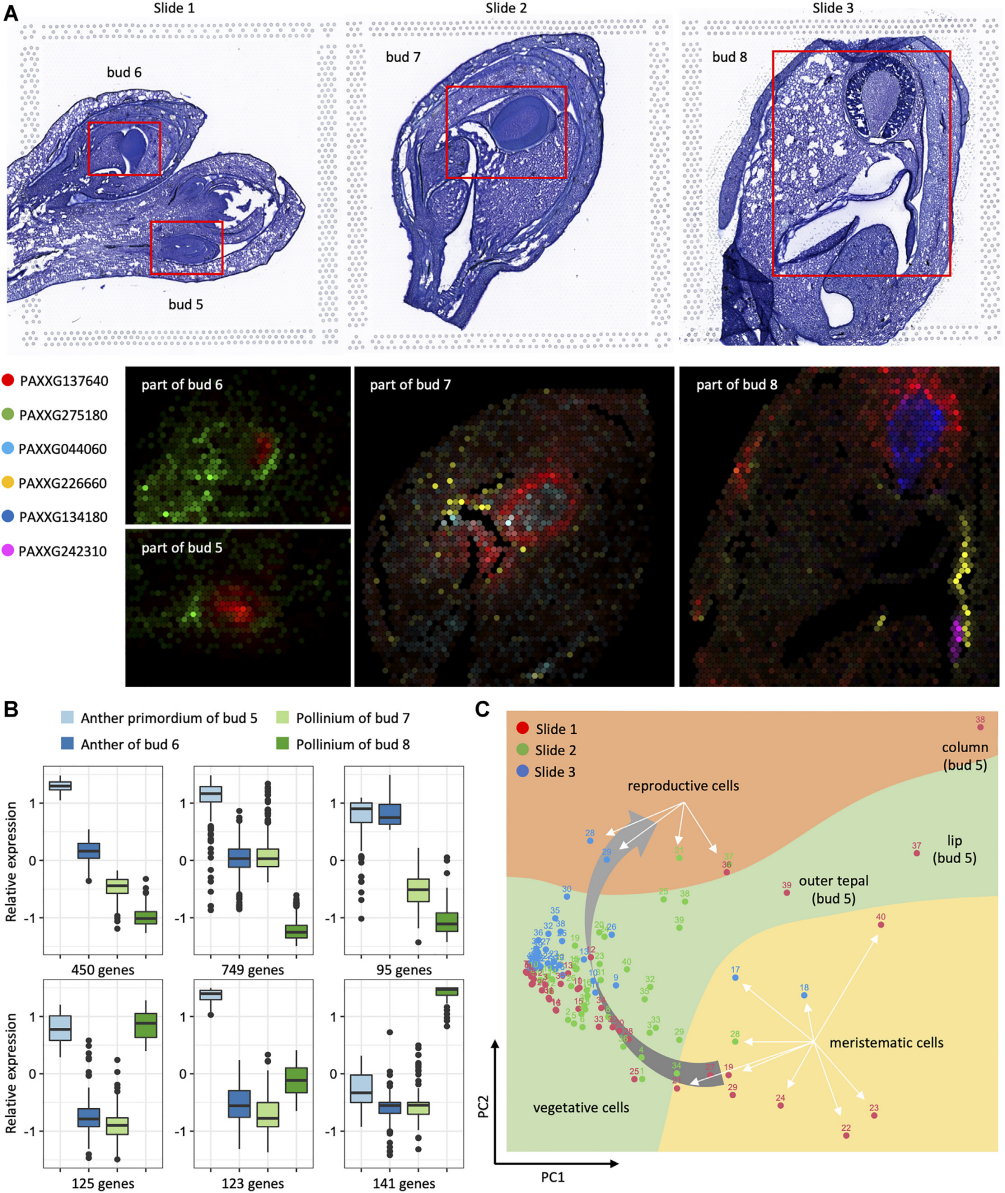
Identity determination and morphogenesis of anther during multiple developmental stages. (**A**) Spatial expression of five marker genes for recognition of anther primordium, pollinium, apex of anther, rostellium and viscidium. (**B**) Comparison of expression levels of 1683 genes preferentially expressed in anther of different stages. (**C**) PCA analysis based on expression similarities among 120 detected cell-types recognized as meristematic, vegetative and reproductive cells.

Unlike vegetative tissues, which could be recognized earlier in floral development, the column structure of orchid flower, also known as gynostemium functioning for reproductivity, is morphologically visible in bud 5 (Supplemental Figure S1), when cells on the top of column are identified as anther primordia, indicated by an individual spot exhibiting peak expression of both B- and C-function genes (Figure 2A). Meanwhile, many downstream genes are activated for further processes of morphogenesis development, e.g. pollinium cells appear on the center of anther in bud 6, and the shape-shifting processes are observed at buds 7 and 8 (Figure 5A). To further explore the dynamic changes of gene expression during anther development, 1683 genes preferentially expressed on anther primordium or pollinium from bud 5 to bud 8 are collected for comparison, resulting in various patterns among different buds. As shown in Figure 5B, 123 and 141 genes are highly expressed in bud 5 and 8, respectively. While the majority of them (the three upper panels with 1294 genes, 76.9%) exhibit an overall decreasing expression pattern from bud 5 to bud 8. Among these genes, the group in the middle chart, with 749 genes, consists of many genes that are essential for reproduction. For example, PAXXG041370 (homologous to *AtRAD50*) plays key roles in prophase I of early meiotic processes in *A. thaliana* (50). PAXXG137640 (homologous to *AtKHZ1*), coding for C3H zinc finger protein responsible for transcription regulation and leading to phenotype of late flowering if mutated in *A. thaliana* (51), is strongly expressed in all column cells but even higher in anther primordium in bud 5, and shows median expression level in core area of anther of bud 6 and continuously expresses in pollinium, rostellum and lip projection area in bud 7, and then withdraws to pollinium of bud 8 with relatively lower expression levels (Figure 5A).

Figure 5 illustrates a series of key genes which are either specially or preferentially expressed in reproduction-related vegetative cells of orchid flower, displaying a dynamic change of the expression profilings during anther development. PAXXG275180, expressed in outer layer of anther, functioning in cell wall modification, shows high similarity in gene expression to tissues of column (bud 6 of Figure 5A) and is likely to be originated from basal meristematic cells (bud 5 of Figure 5A). Upon the formation of the outer and inner vegetative tissues which encircle the pollinium, many genes are observed specifically expressed in these regions, e.g. PAXXG226660 for apex of anther and tegula (buds 7 and 8), PAXXG242310 (GDSL-like Lipase in *A. thaliana* (52)) for viscidium, PAXXG044060 (chlorophyll A/B binding protein (53)) and PAXXG134180 for inner cells but expressed at different stages. As show in Figure 5C, PCA analysis upon the 120 clusters of the three samples further supports the observation that vegetative tissues in anther (cluster 25 of slide 2, clusters 30 and 35 of slide 3) exhibit high gene expression similarity to pollinium (cluster 21 of slide 2, clusters 28 and 29 of slide 3). These results suggest that the early vegetative tissues of anther, though often sharing gene expressions rather than exhibiting specifically expressed genes, undergo processes of differentiation and morphogenesis rapidly.

### Gene duplications increasing complexity of transcription regulation during flower development

Gene duplicates usually experience functional divergence by accumulation of mutations or altering gene dosage balance, thus providing evolutionary potentials for development, environmental adaptation and biodiversity (54–56). Angiosperms have undergone multiple polyploidy events on their common ancestor and on many lineages with high biodiversity including Orchidaceae (57–60), leaving vast amounts of gene duplicates in their genomes. Similar to the MADS-box gene family, many of which are essential for identity determination of floral organs (Figure 3), further phylogenomic analysis reveals spatiotemporal expression variation for many other gene families exhibiting ancient and recent duplication events, e.g. auxin response factor (ARF), AUX/IAA, auxin inducible protein, cytokinin oxidase, cytokinin-responsive gata factor, bHLH and MYB (Supplementary Figure S14-S20). Among them, the basic/helix-loop-helix (bHLH) proteins are one of the largest families of transcriptional regulators in eukaryotes. In plants, bHLH transcription factors play key roles in diverse biological processes, including photomorphogenesis, floral induction, secondary metabolite biosynthesis, biotic and abiotic stress responses, etc. (61). Among the 115 bHLH genes of *P. aphrodite*, 49 of them are expressed in spatial transcriptomes of *P*. Big Chili (with five or more reads detected on at least one spot) among meristematic, vegetative and/or reproductive tissues across different stages. For example, PAXXG122980 (homologous to *AtKDR* (62)) specifically expressed in bilaterally symmetrical meristematic tissues of both buds 5 and 6 (Supplementary Figure S21), while the other two paralogs (PAXXG030200 and PAXXG239050) yielded from a duplication event after divergence of angiosperm exhibiting relatively weak expression signals suggesting they have undergone functional divergence. Similar observations are found for another subfamily of bHLH: PAXXG325470 is preferential in deep meristematic tissues of bud 5 and symmetrical meristematic cells of bud 6 (Supplementary Figure S21), but the expression area of PAXXG239600 covers all floral organs.

MYB genes, another superfamily of transcription factors, have been well characterized in eukaryotes as being involved in the regulation of various biological processes including development, differentiation, environmental adaptation and metabolic regulation in plants (63). There are 25 MYB genes of *P*. Big Chili (135 genes in total in *P. aphrodite*) detected in the three spatial transcriptomic datasets. Genes duplicated after divergence of angiosperms, though exhibiting high sequence similarities, already present divergence on varying tissues or developing stages. In particular, PAXXG071610 shows preferential expression in meristems, tepals and lips of both early and late stage buds, while PAXXG290370 is enriched in meristems of early stage buds and also in tepals of late stage buds (Supplemental Figure S21). Despite of functional divergence of duplicated genes, protein interactions of multiple gene families further enhance complexities on transcription regulation, e.g. protein complexes consisting of members of MYB, bHLH, and WD40 families are proposed to regulate cell differentiation during anthocyanidin, proanthocyanin, and seed-coat mucilage, and the development of trichomes and root hairs (64).

## DISCUSSION

In this study, we analyzed spatial transcriptomic datasets for investigating dynamic changes of gene expressions functioning in early development of floral organs of *P*. Big Chili. By comparison of spatiotemporal expression distributions, thousands of genes preferentially expressed in specific tissues/stages are identified, including well-known MADS-box genes and many other potential downstream genes, yielding a model of floral organ initialization and identity determination (Figure 5C). Our study presents a system-wide survey of flowering development of orchid and provides valuable resources for further investigation of gene regulatory networks underlying the complex but essential process.

Upon the sequencing information of all individual beads/spots with spatiotemporal contexts, we can identify specific cell-types or tissues according to the gene expression profilings, or vice versa. As illustrated in Figure 3, the specific expression of PAXXG198660 and PAXXG301780 of AGL6 family in lip and tepals, respectively, provide evidences for their roles in identity determination of corresponding organs. More importantly, mutants of many genes of plants lack visible phenotypes, especially for the duplicated genes having both redundant and divergent functions. The combination of spatial transcription information of given genes, phylogenetic relationship of related gene families and co-expression networks analyses may provide valuable priori information for understanding gene functions, even when morphology of mutants are nearly normal.

Compared with standard bulk RNA-seq of small flower bud of orchid, the spatial transcriptome in this study detects similar amounts of transcribed genes with consistent transcription levels (Supplementary Figure S22), suggesting the application of this technology would greatly benefit the study on plant. Nevertheless, challenges still remain in procedures of sample preparation when applying spatial transcriptome sequencing on plant tissues. As it takes hours for tissue fixation, embedding and cryosection, determining RNA integrity is a critical factor for downstream RNA amplification and parallel sequencing. In addition, plant cells differ widely in size and shape, bringing large variations in the amount of RNA molecules captured by beads/spots of same size. Therefore, there is a need for the development of computational algorithms for cell clustering by integration the information of cell size and shape of histological images.

## DATA AVAILABILITY

All spatial transcriptomic data of orchid flower samples are available at NCBI Gene Expression Omnibus with accession number GSE198128, and also deposited at Open Science Framework (https://osf.io/mqsb4/?view_only=787b3d5a81e54c27b3f33d5841b59a51).

## Supplementary Material

gkac773_Supplemental_Files
