# Supplementary material for: A spatiotemporal atlas of organogenesis in the development of orchid flowers
Source: Nucleic Acids Res. 2022 Sep 12;50(17):9724–37. doi: 10.1093/nar/gkac773 (PMC9508851; doi:10.1093/nar/gkac773)
Supplement: gkac773_Supplemental_Files [file gkac773_supplemental_files.zip › Supplementary_Figure_S14-20.pdf]

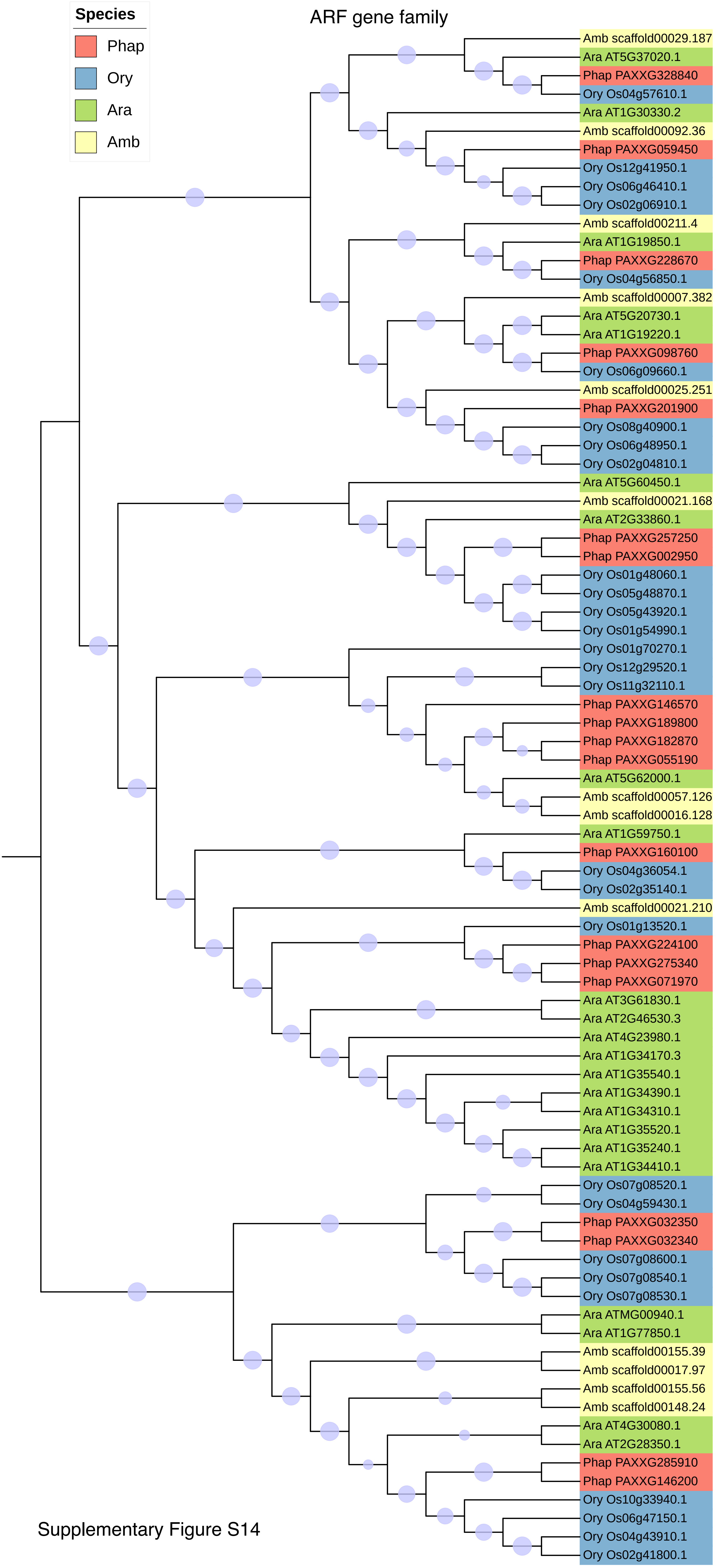

Supplementary Figure S14

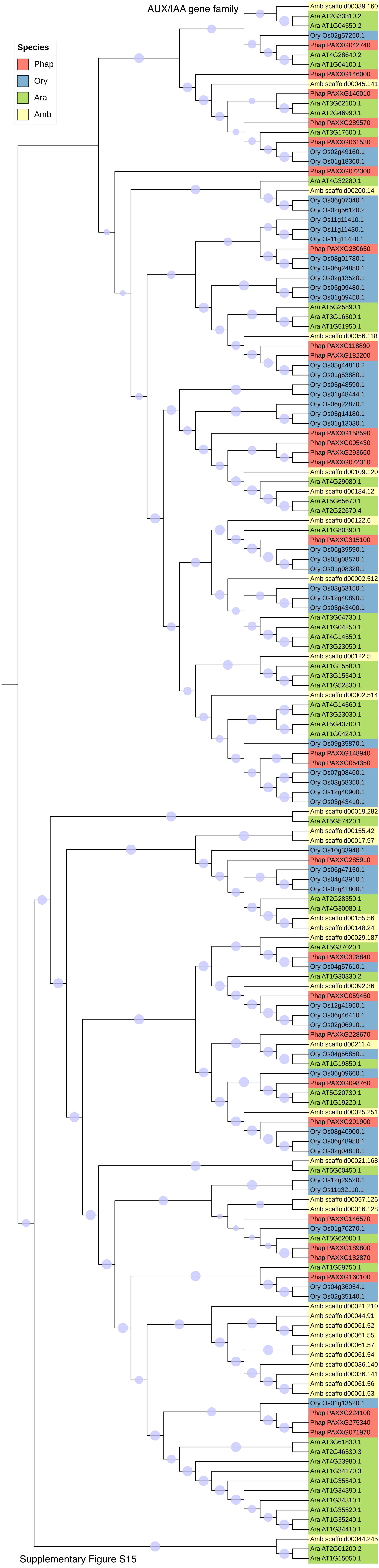

Supplementary Figure S15

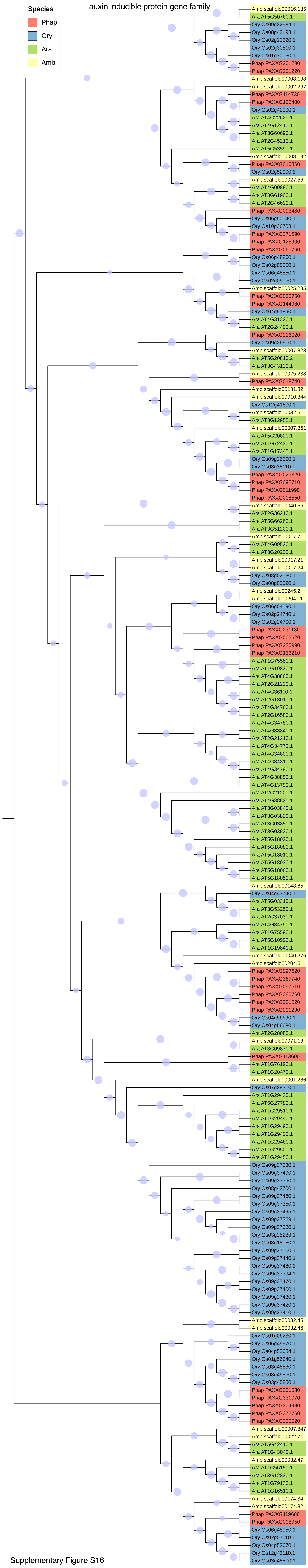

Supplementary Figure S16

cytokinin oxidase gene family

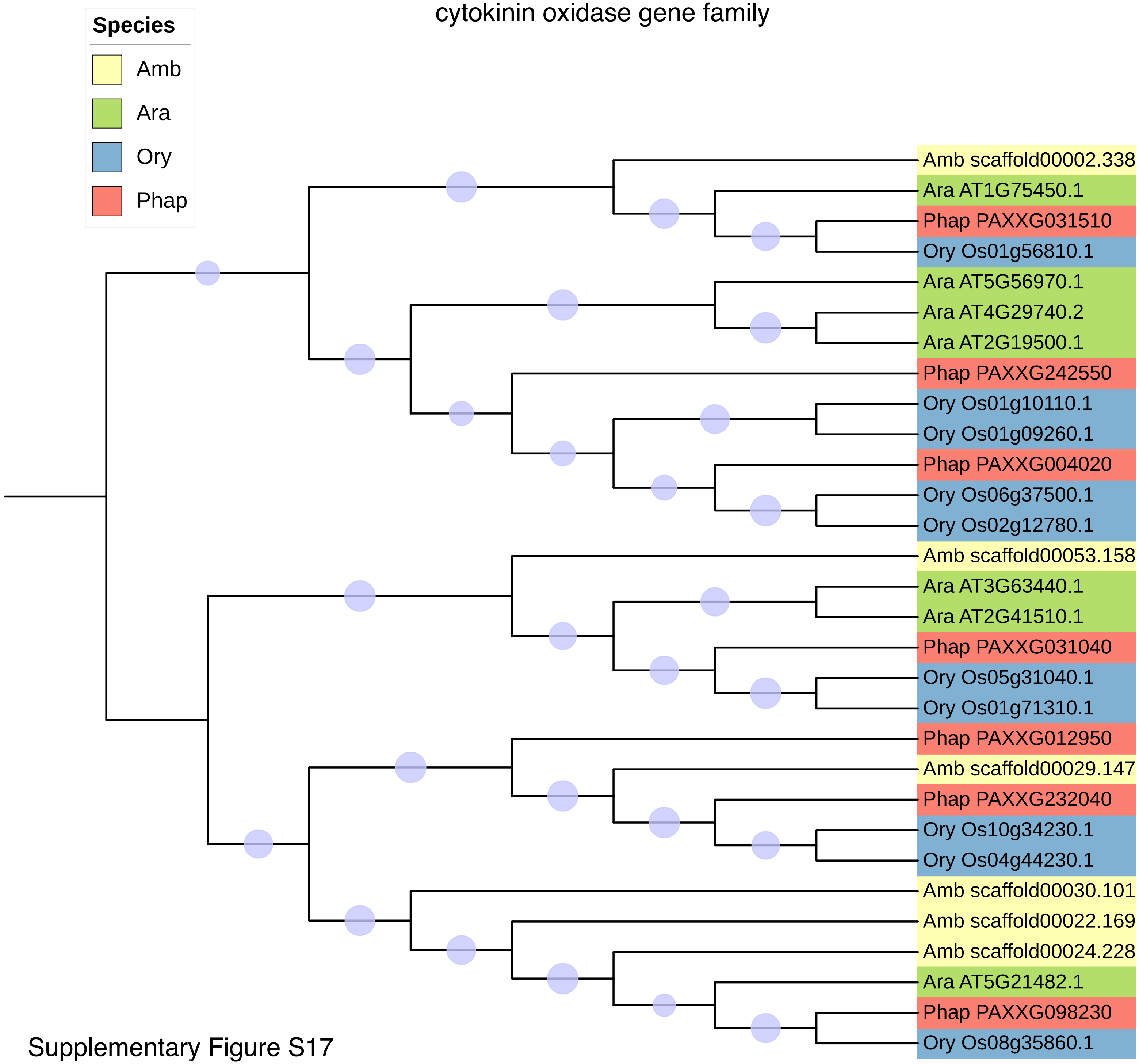

Supplementary Figure S17

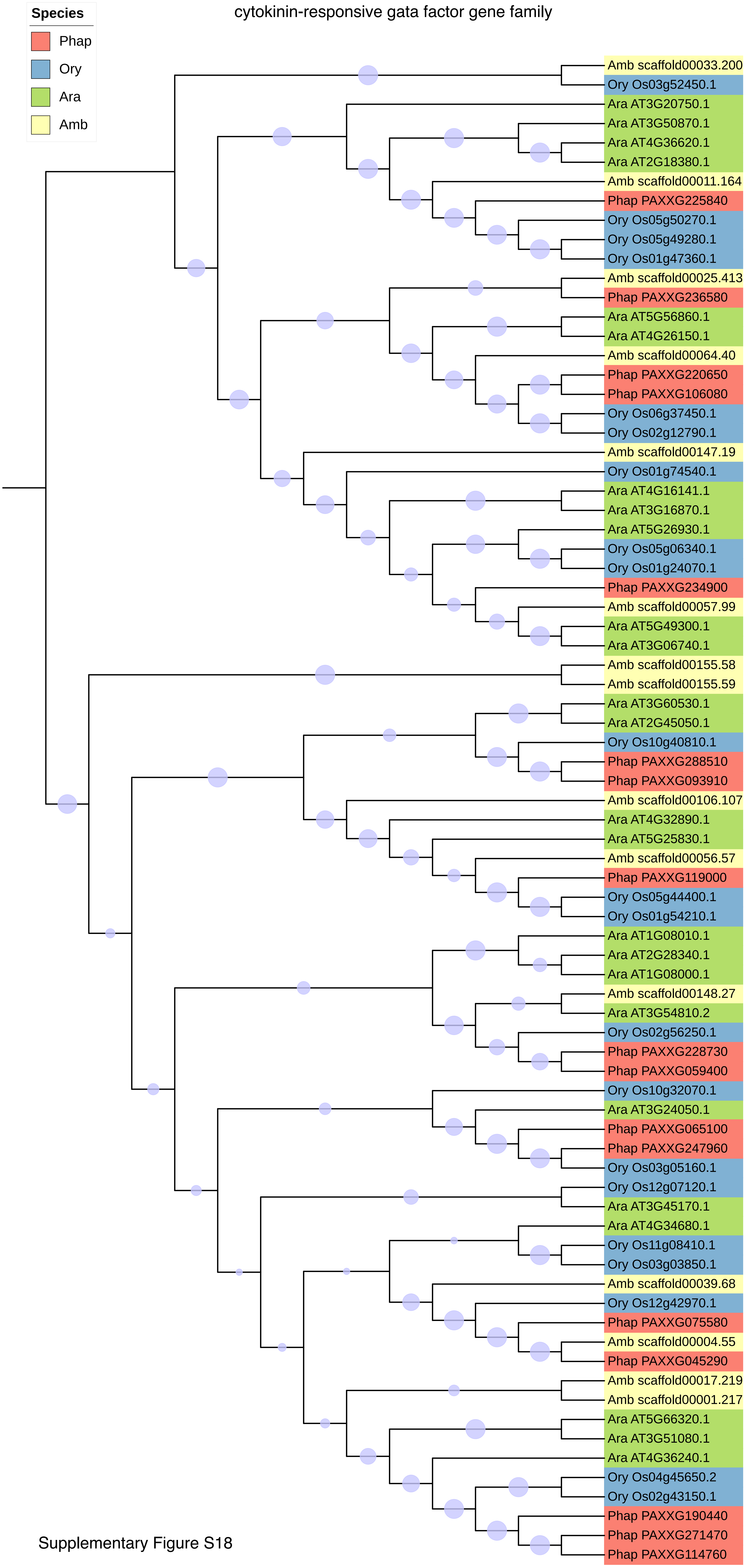

Supplementary Figure S18

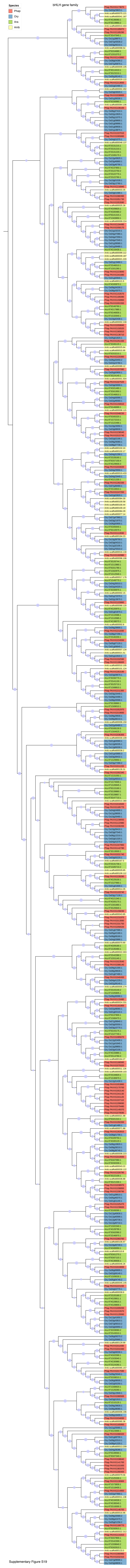

Supplementary Figure S19

MYB gene family

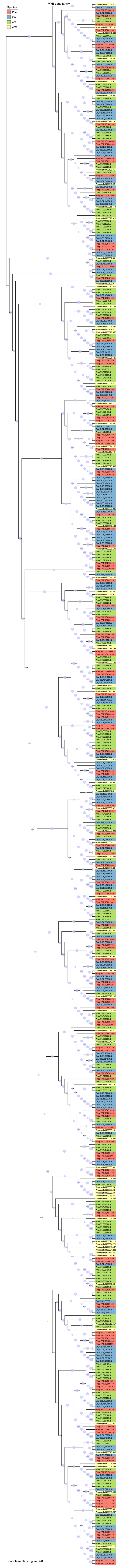

Supplementary Figure S20
