# Supplementary material for: A spatiotemporal atlas of organogenesis in the development of orchid flowers
Source: Nucleic Acids Res. 2022 Sep 12;50(17):9724–37. doi: 10.1093/nar/gkac773 (PMC9508851; doi:10.1093/nar/gkac773)
Supplement: gkac773_Supplemental_Files [file gkac773_supplemental_files.zip › supporting_information_20220817.pdf]

Supporting Information

Supplementary Table S1. A summary of three spatial transcriptomic datasets sequenced in this study.

Supplementary Table S2. A list of marker genes preferentially expressed in various tissues of slide 1.

Supplementary Table S3. A list of marker genes preferentially expressed in various tissues of slide 2.

Supplementary Table S4. A list of marker genes preferentially expressed in various tissues of slide 3.

Supplementary Figure S1. Presentation of Trypan blue stain image for early development stages of orchid flowers (dataset 1).

Supplementary Figure S2. Application of STEEL for cell type detection on the sample of dataset 1 sequenced on 10X Visium platform (40 groups, as shown in Figure 1B).

Supplementary Figure S3. Hierarchical clustering of 40 cell types identified by STEEL on the sample of dataset 1.

Supplementary Figure S4. Presentation of Trypan blue stain image for early development stages of orchid flowers (dataset 2).

Supplementary Figure S5. Application of STEEL for cell type detection on the sample of dataset 2 sequenced on 10X Visium platform (40 groups, as shown in Figure 1B).

Supplementary Figure S6. Hierarchical clustering of 40 cell types identified by STEEL on the sample of dataset 2.

Supplementary Figure S7. Presentation of Trypan blue stain image for early development stages of orchid flowers (dataset 3).

Supplementary Figure S8. Application of STEEL for cell type detection on the sample of dataset 3 sequenced on 10X Visium platform (40 groups, as shown in Figure 1B).

Supplementary Figure S9. Hierarchical clustering of 40 cell types identified by STEEL on the sample of dataset 3.

Supplementary Figure S10. Gene Ontology enrichment for the six clusters of genes in Figure 2D. Top 20 most enriched GO terms for each cluster are presented.

Supplementary Figure S11. Illustration of spatial expression of selected genes related to auxin and cytokinin, grouped according to preferential tissues.

Supplementary Figure S12. A maximum likelihood tree of MADS-box homologs of seven angiosperm species.

Supplementary Figure S13. Gene Ontology enrichment for the four clusters of genes in Figure 4C. Top 20 most enriched GO terms for each cluster are presented.

Supplementary Figure S14. A maximum likelihood tree of auxin response factor (ARF) gene family of four angiosperm species.

Supplementary Figure S15. A maximum likelihood tree of AUX/IAA gene family of four angiosperm species.

Supplementary Figure S16. A maximum likelihood tree of auxin inducible protein gene family of four angiosperm species.

Supplementary Figure S17. A maximum likelihood tree of cytokinin oxidase gene family of four angiosperm species.

Supplementary Figure S18. A maximum likelihood tree of cytokinin-responsive gata factor gene family of four angiosperm species.

Supplementary Figure S19. A maximum likelihood tree of bHLH gene family of six angiosperm species. The bootstrap values with 100 replicates are given for each node on the tree.

Supplementary Figure S20. A maximum likelihood tree of MYB gene family of six angiosperm species.

Supplementary Figure S21. Illustration of spatial expression of selected genes of bHLH and MYB gene families on different flowering stages.

Supplementary Figure S22. Comparison of gene expression values of small flower bud detected by *P. aphrodite* by bulk RNA-seq (Chao et al. 2017, Plant Cell Physiology, NCBI GEO: SRR4302012) and by spatial transcriptome sequencing (bud 8).

Supplementary Table S1. A summary of three spatial transcriptomic datasets sequenced in this study. The detailed description of each metric is available at 10X Genomics (https://support.10xgenomics.com/single-cell-gene-expression/software/pipelines/latest/output/gex-metrics)

Gene Expression Metrics	Slide 1	Slide 2	Slide 3
Sequencing			
Number of Reads	199,680,090	198,604,175	194,608,936
Valid Barcodes	91.00%	91.40%	91.00%
Valid UMIs	100.00%	100.00%	100.00%
Sequencing Saturation	71.00%	66.20%	75.50%
Q30 Bases in Barcode	96.00%	95.90%	96.00%
Q30 Bases in RNA Read	90.50%	91.10%	90.40%
Q30 Bases in UMI	92.80%	93.10%	93.30%
Mapping			
Reads Mapped to Genome	85.70%	86.90%	86.50%
Reads Mapped Confidently to Genome	80.40%	81.70%	80.90%
Reads Mapped Confidently to Intergenic Regions	22.70%	22.90%	25.60%
Reads Mapped Confidently to Intronic Regions	7.30%	8.10%	7.10%
Reads Mapped Confidently to Exonic Regions	50.40%	50.70%	48.30%
Spots			
Number of Spots Under Tissue	2,379	2,467	3,407
Fraction Reads in Spots Under Tissue	84.50%	68.60%	80.80%
Mean Reads per Spot	83,934	80,504	57,120
Mean Reads Under Tissue per Spot	53,629	47,352	39,403
Median UMI Counts per Spot	3,675	5,811	2,495
Median Genes per Spot	1,951	2,874	1,429
Total Genes Detected	17,486	17,948	17,719

(The following three tables are provided as separate files)

Supplementary Table S2. A list of marker genes preferentially expressed in various tissues of slide 1. For each gene, preferentially expressed cluster(s) measured by STEEL is provided. The best matched homolog in *Arabidopsis thaliana* is listed when aligned sequence owns $\geq$ 40% similarity and occupies $\geq$ 50% region of query/subject gene. MapMan annotation are predicted using Mercator4, GO terms are annotated by eggNOG-mapper.

Supplementary Table S3. A list of marker genes preferentially expressed in various tissues of slide 2.

Supplementary Table S4. A list of marker genes preferentially expressed in various tissues of slide 3.

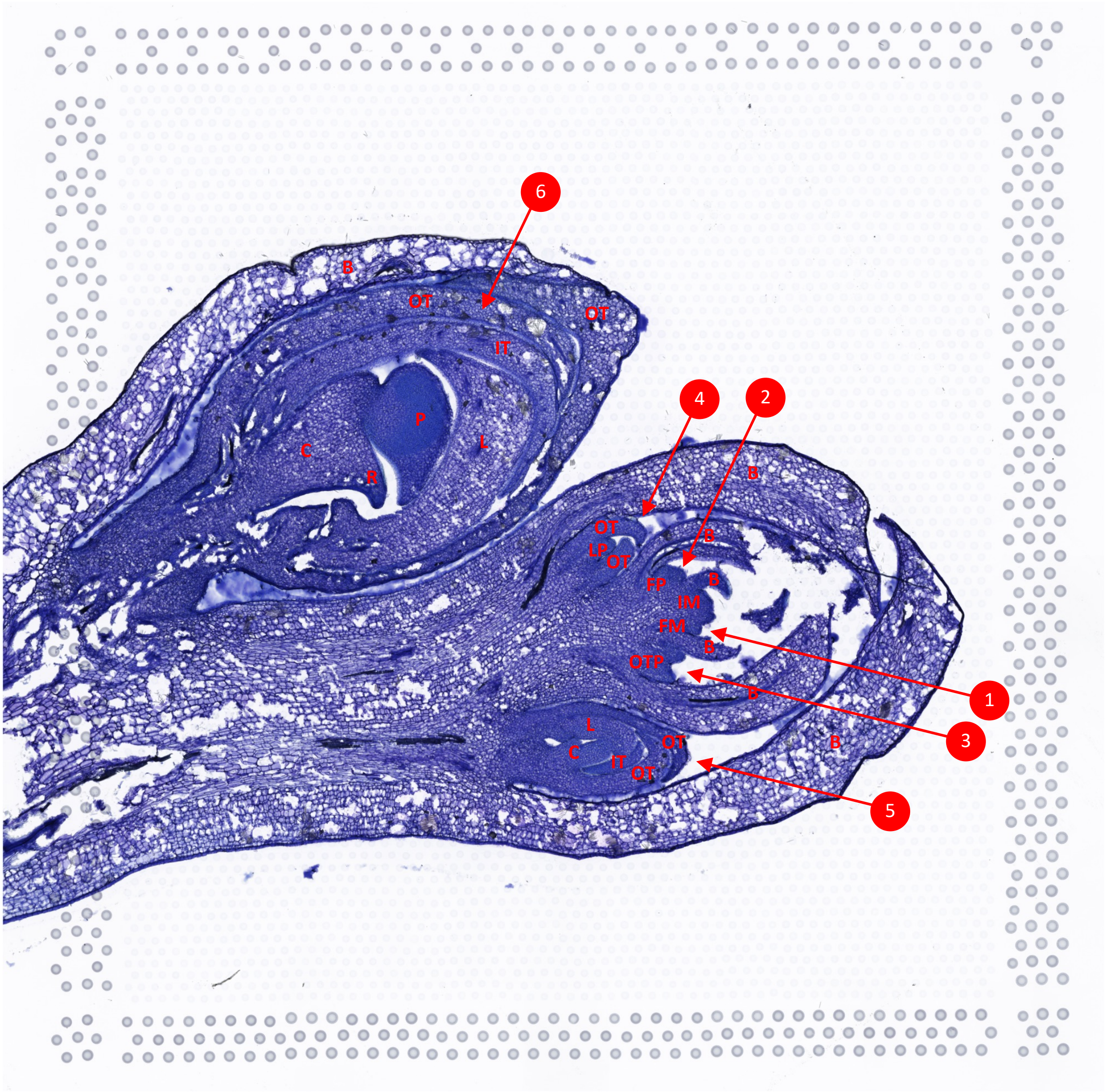

Abbr.	Tissue	Abbr.	Tissue	Abbr.	Tissue	Abbr.	Tissue
B	Bract	OT	Outer Tepal	C	Column	V	Viscidium
IM	Inflorescence Meristem	IT	Inner Tepal	CF	Column foot	T	Tegula
FM	Floral Meristem	L	Lip	P	Pollinium		
FP	Floral Primordium	LP	Lip Primordium	R	Rostellum		
OTP	Outer Tepal Primordium	LPJ	Lip Projection	AOA	Apex Of Anther		

Supplementary Figure S1. Presentation of Trypan blue stain image for early development stages of orchid flowers (dataset 1). Abbreviations of different tissues are listed in the table on the lower panel.

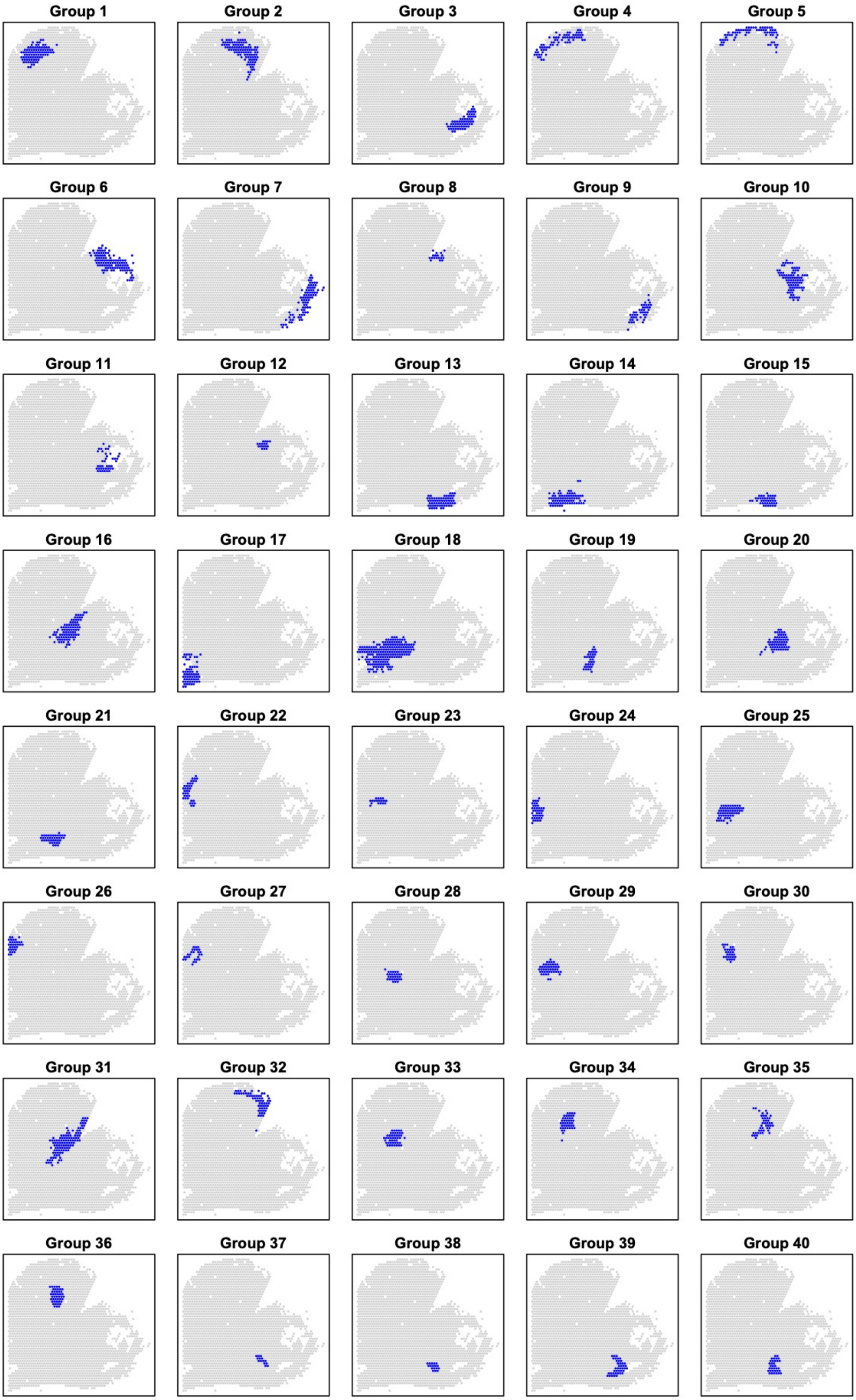

Supplementary Figure S2. Application of STEEL for cell type detection on the sample of dataset 1 sequenced on 10X Visium platform (40 groups, as shown in Figure 1B).

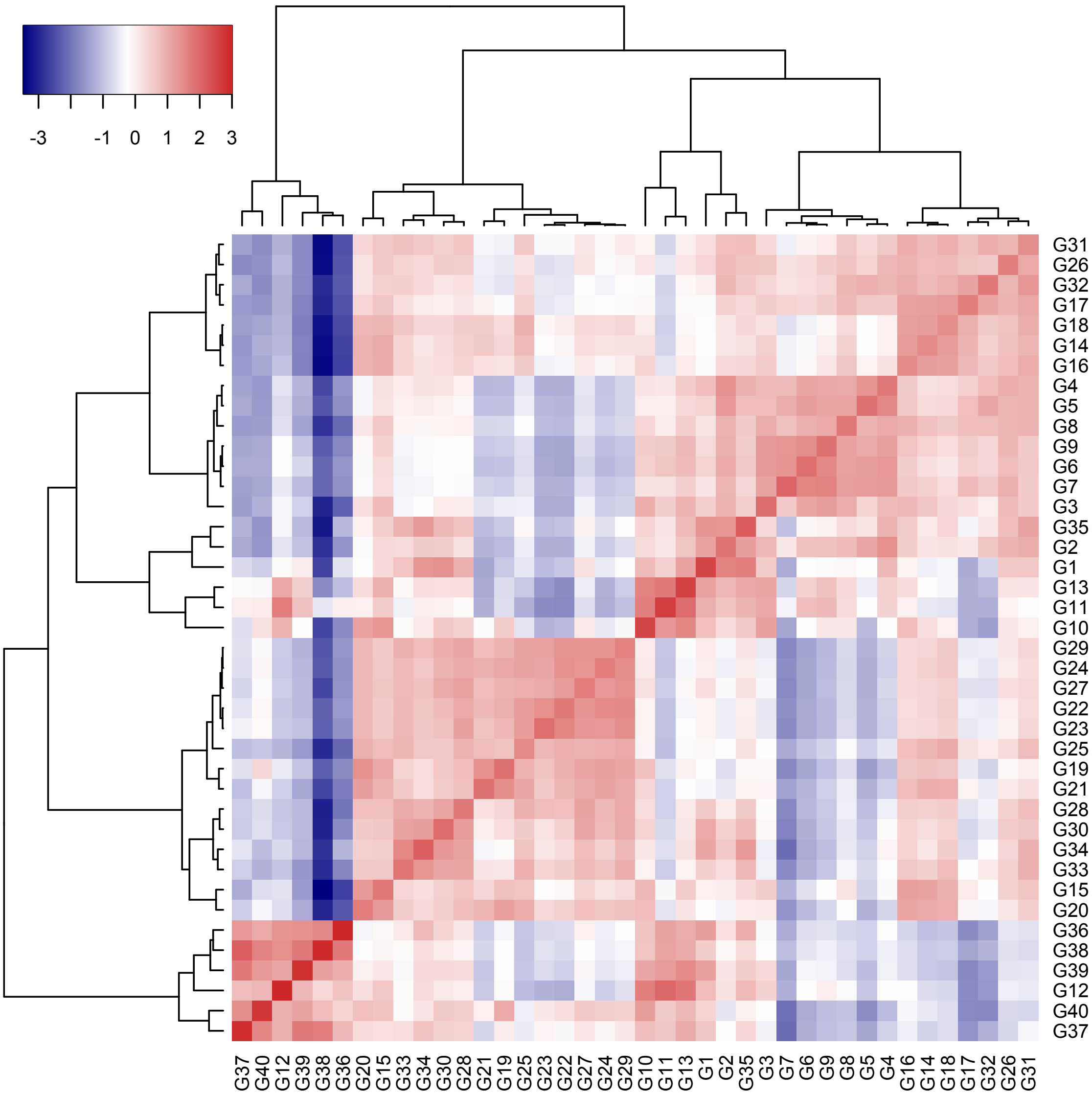

Supplementary Figure S3. Hierarchical clustering of 40 cell types identified by STEEL on the sample of dataset 1.

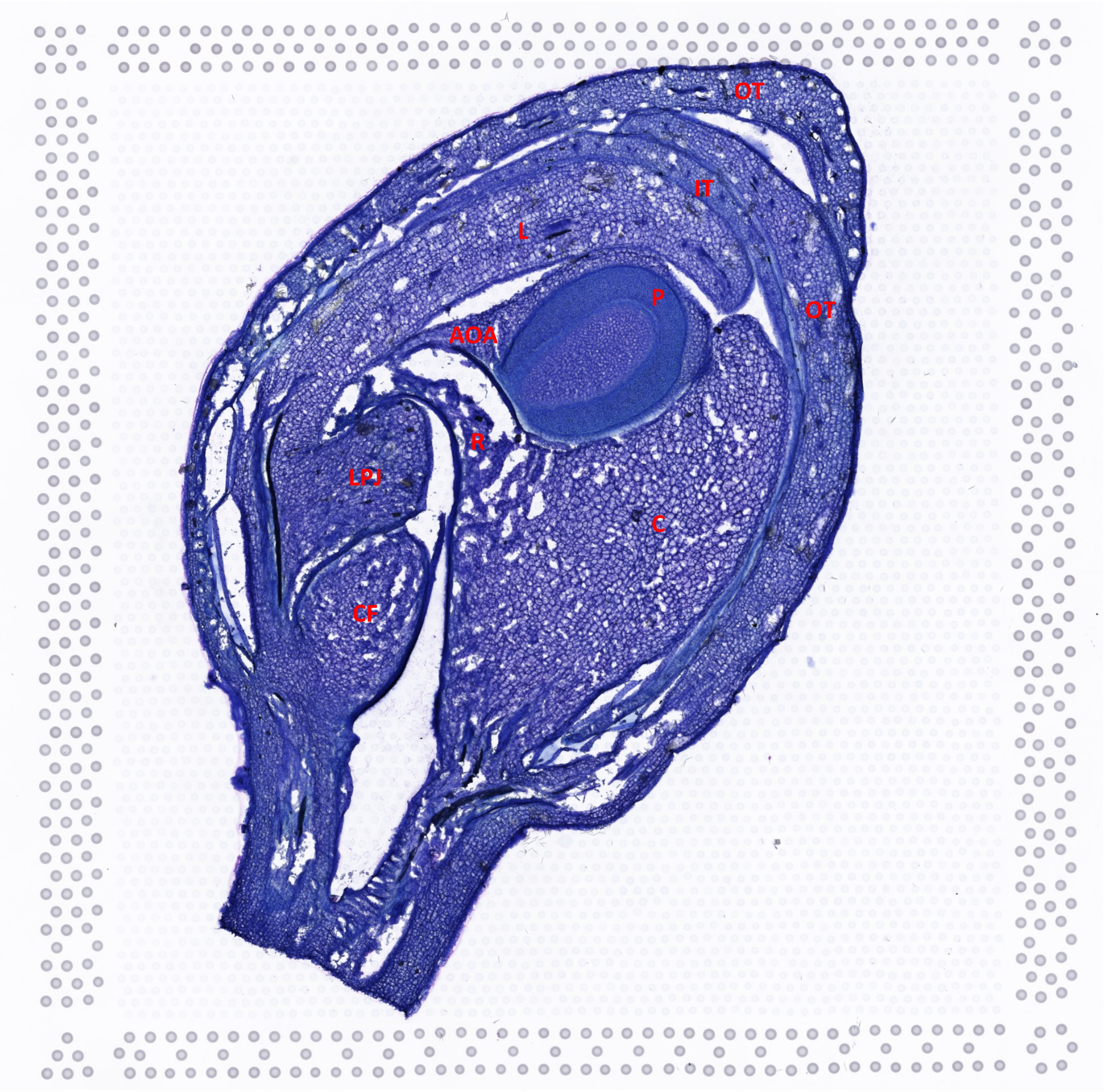

Abbr.	Tissue	Abbr.	Tissue	Abbr.	Tissue	Abbr.	Tissue
B	Bract	OT	Outer Tepal	C	Column	V	Viscidium
IM	Inflorescence Meristem	IT	Inner Tepal	CF	Column foot	T	Tegula
FM	Floral Meristem	L	Lip	P	Pollinium		
FP	Floral Primordium	LP	Lip Primordium	R	Rostellum		
OTP	Outer Tepal Primordium	LPJ	Lip Projection	AOA	Apex Of Anther		

Supplementary Figure S4. Presentation of Trypan blue stain image for early development stages of orchid flowers (dataset 2). Abbreviations of different tissues are listed in the table on the lower panel.

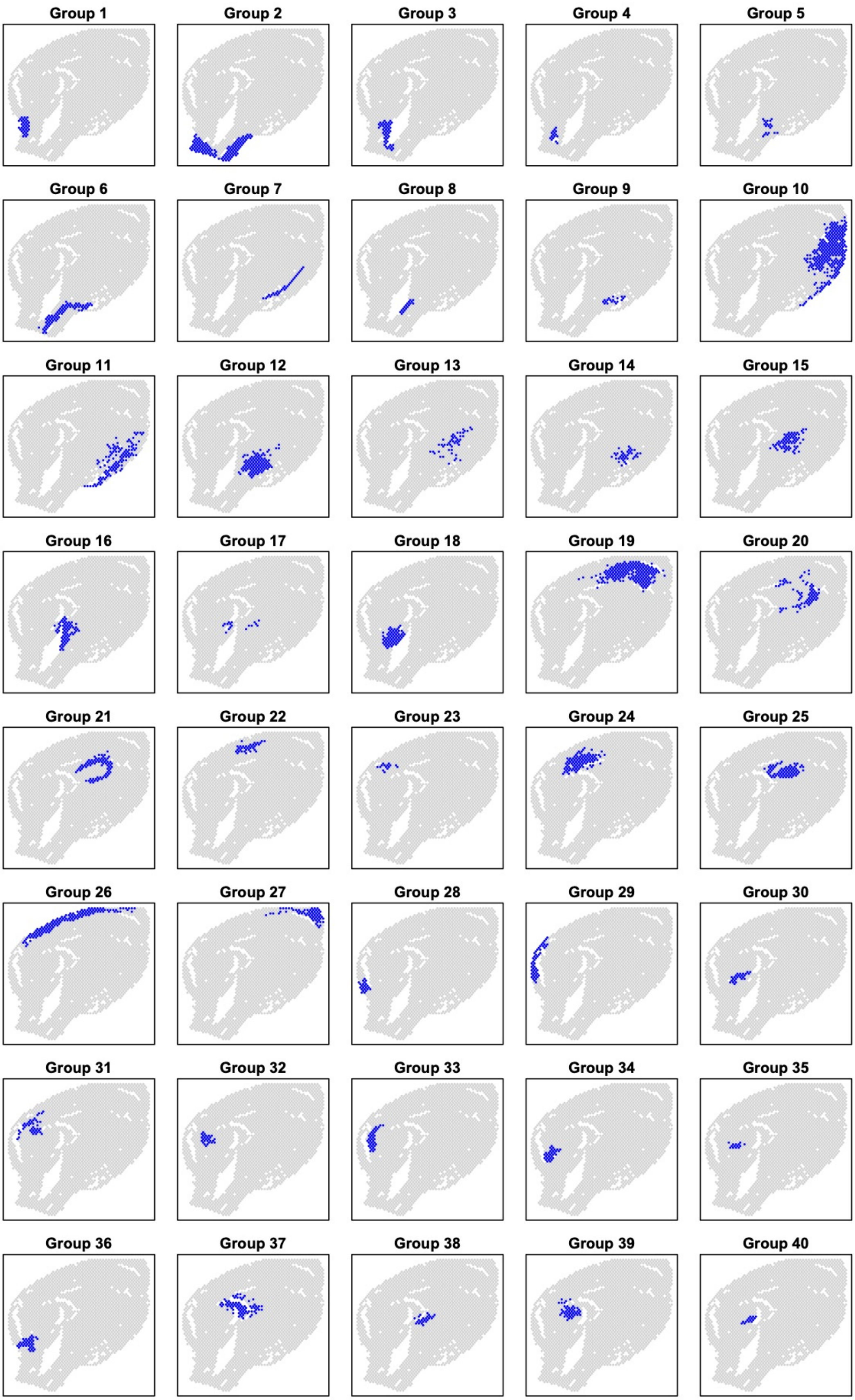

Supplementary Figure S5. Application of STEEL for cell type detection on the sample of dataset 2 sequenced on 10X Visium platform (40 groups, as shown in Figure 1B).

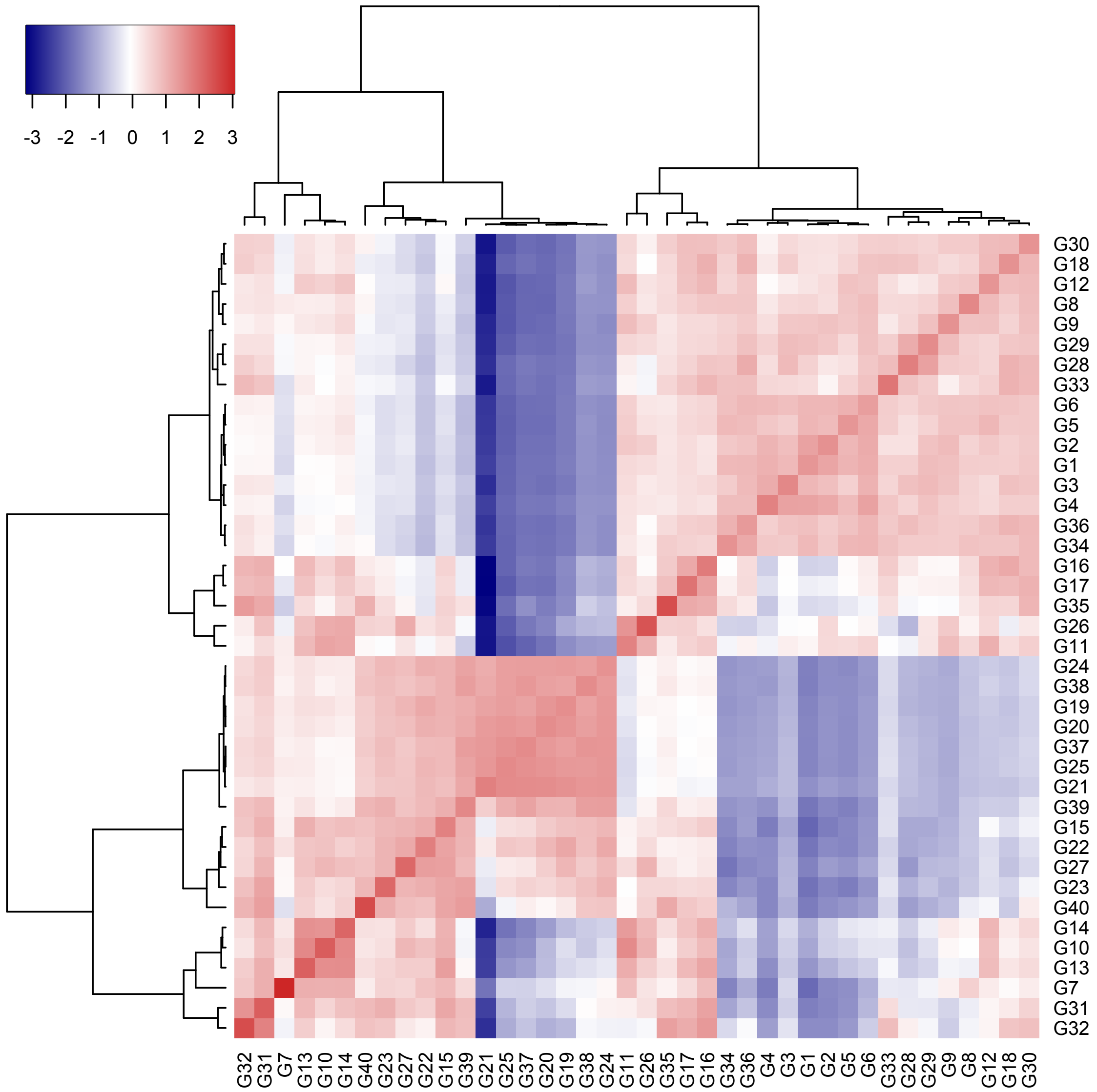

Supplementary Figure S6. Hierarchical clustering of 40 cell types identified by STEEL on the sample of dataset 2.

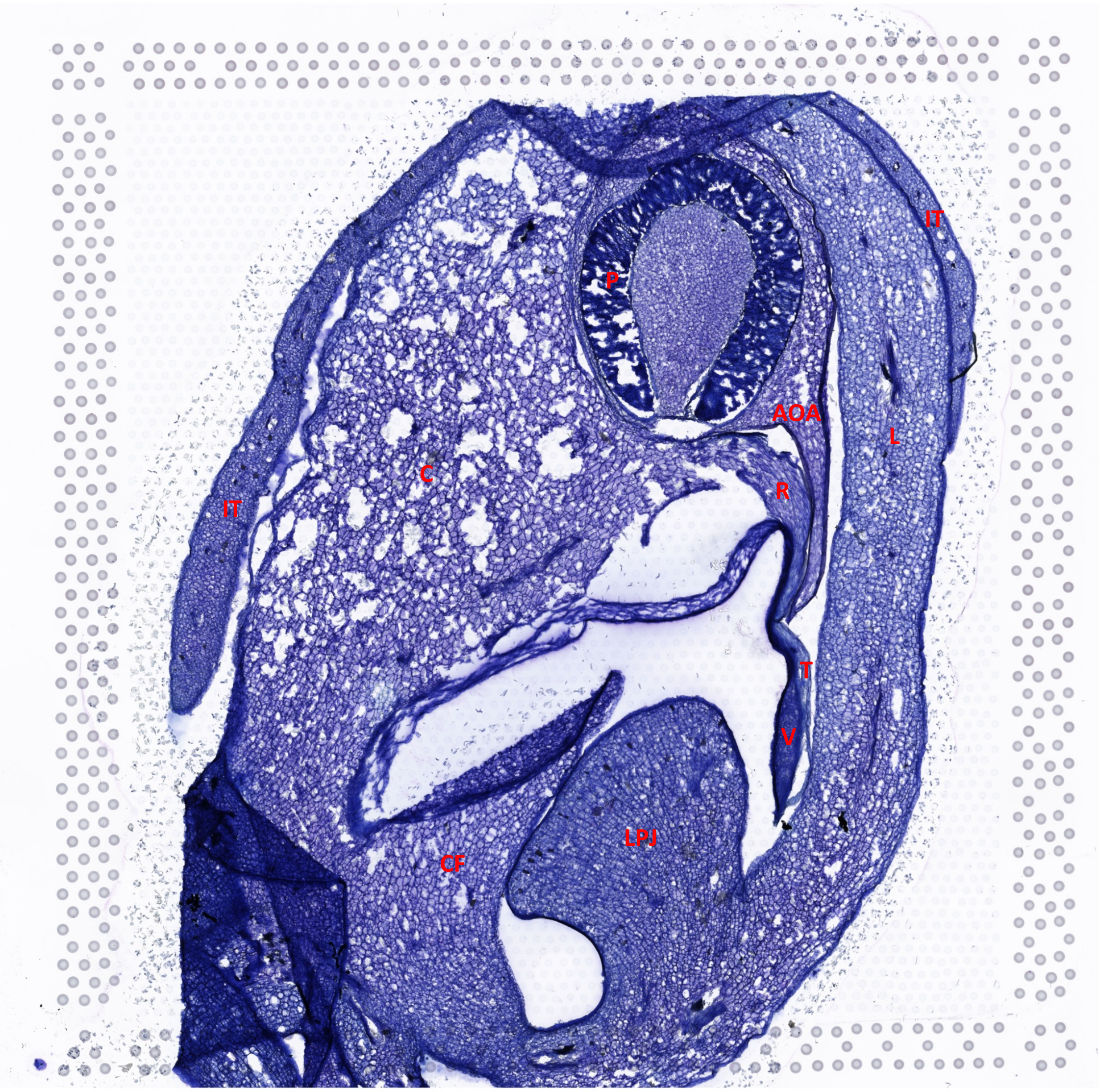

Abbr.	Tissue	Abbr.	Tissue	Abbr.	Tissue	Abbr.	Tissue
B	Bract	OT	Outer Tepal	C	Column	V	Viscidium
IM	Inflorescence Meristem	IT	Inner Tepal	CF	Column foot	T	Tegula
FM	Floral Meristem	L	Lip	P	Pollinium		
FP	Floral Primordium	LP	Lip Primordium	R	Rostellum		
OTP	Outer Tepal Primordium	LPJ	Lip Projection	AOA	Apex Of Anther		

Supplementary Figure S7. Presentation of Trypan blue stain image for early development stages of orchid flowers (dataset 3). Abbreviations of different tissues are listed in the table on the lower panel.

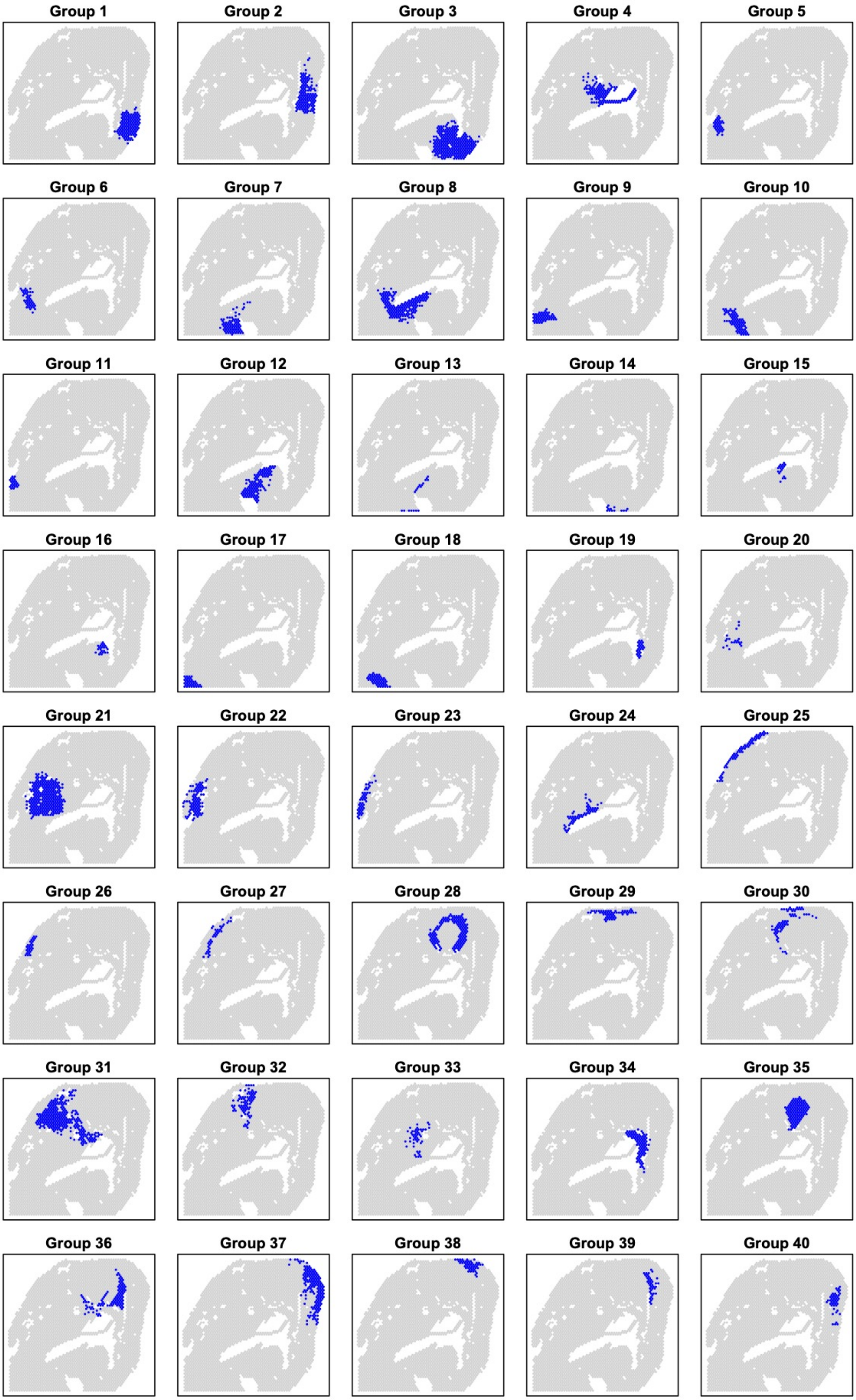

Supplementary Figure S8. Application of STEEL for cell type detection on the sample of dataset 3 sequenced on 10X Visium platform (40 groups, as shown in Figure 1B).

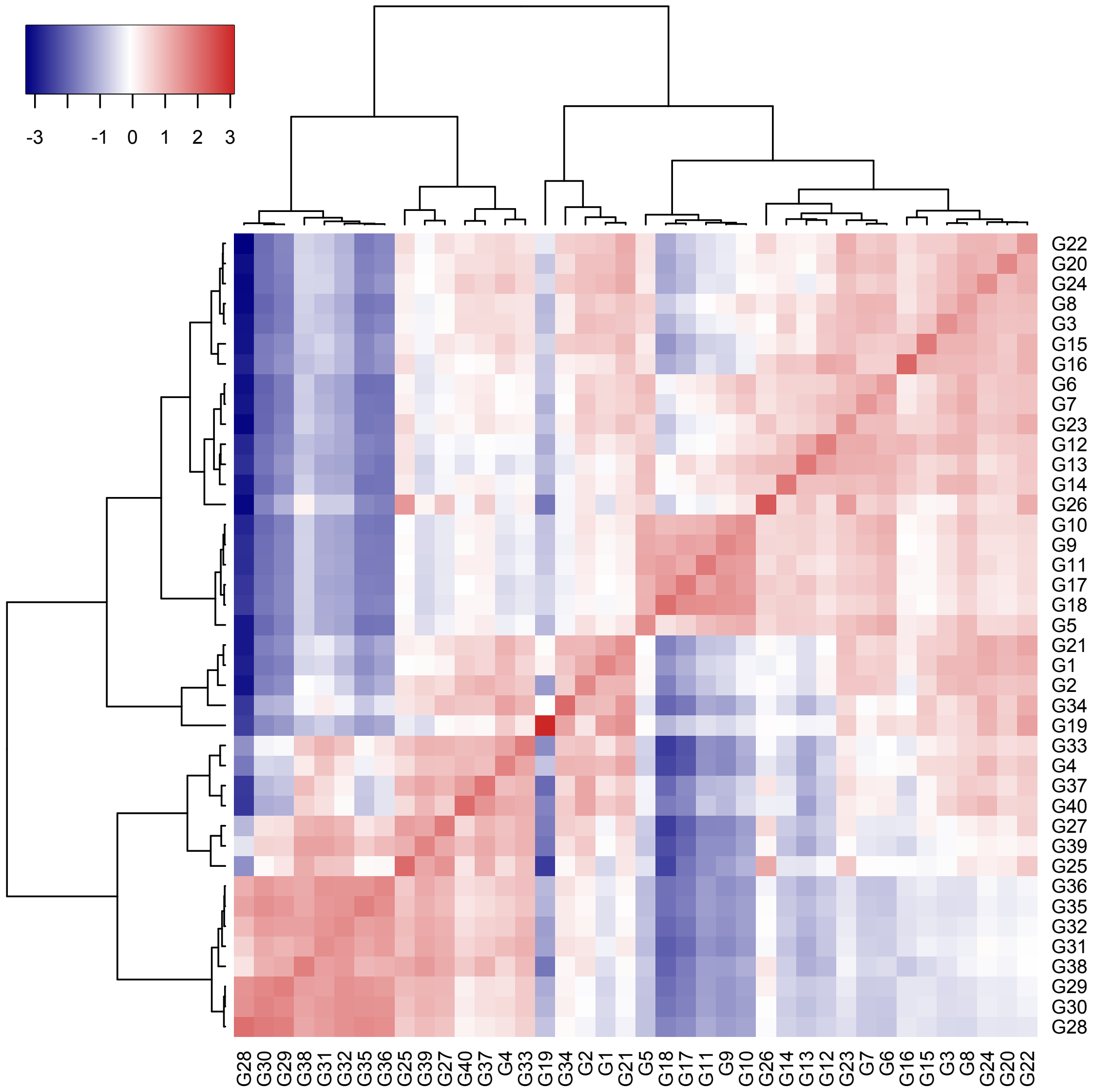

Supplementary Figure S9. Hierarchical clustering of 40 cell types identified by STEEL on the sample of dataset 3.

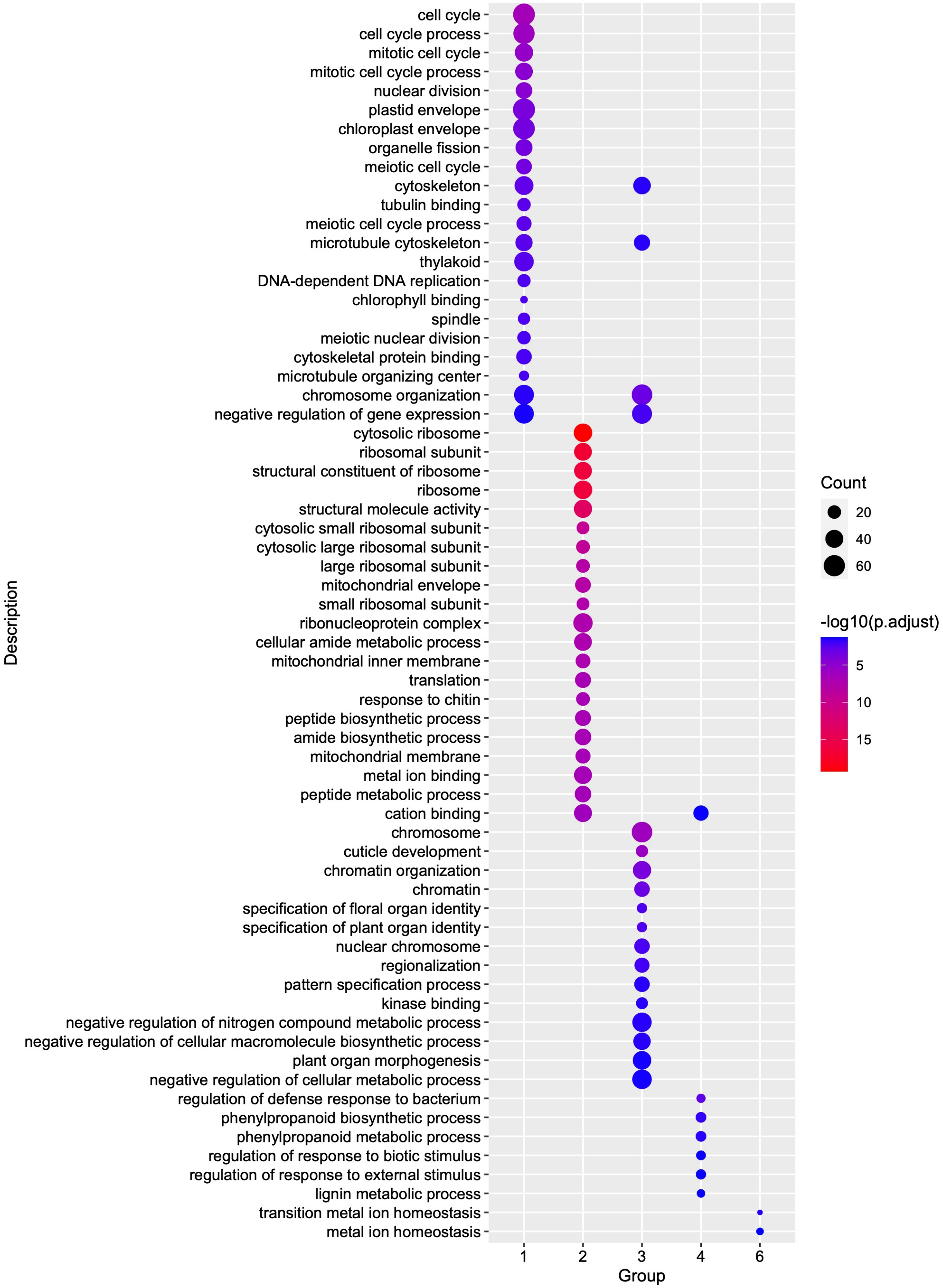

Supplementary Figure S10. Gene Ontology enrichment for the six clusters of genes in Figure 2D. Top 20 most enriched GO terms for each cluster are presented. Please note that no GO term is enriched for genes in cluster 5.

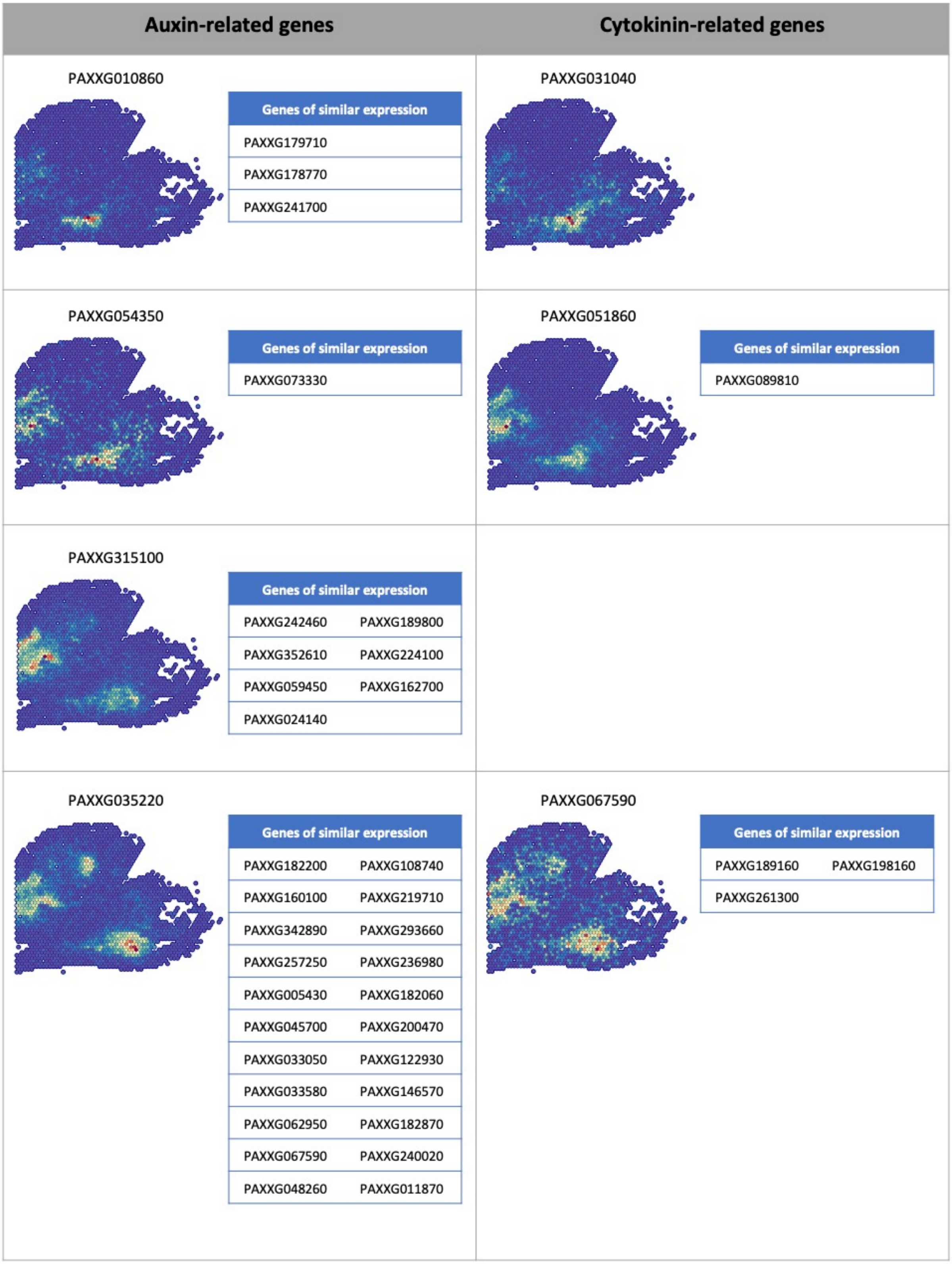

Supplementary Figure S11. Illustration of spatial expression of selected genes related to auxin and cytokinin, grouped according to preferential tissues.

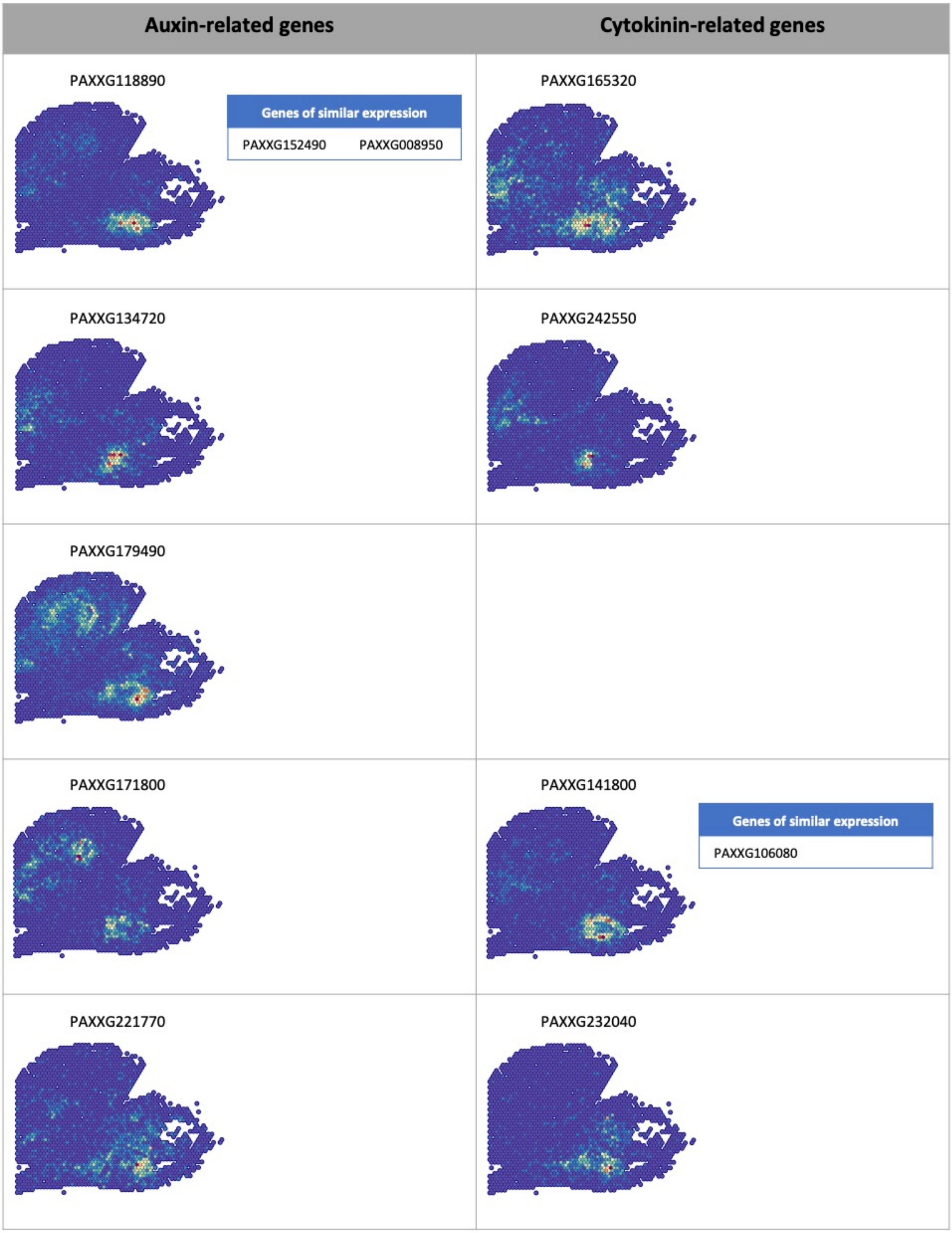

Supplementary Figure S11 (continued). Illustration of spatial expression of selected genes related to auxin and cytokinin, grouped according to preferential tissues.

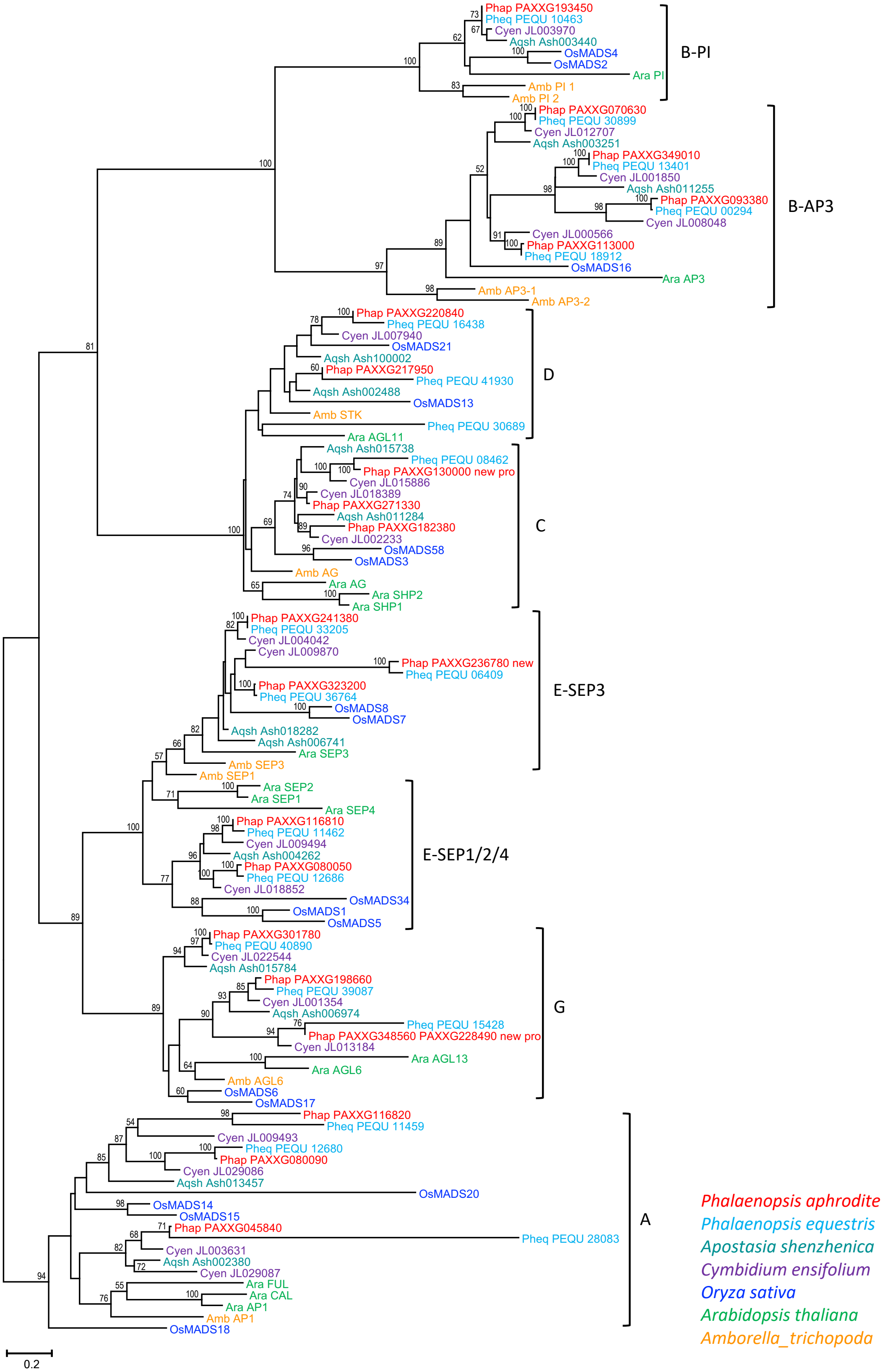

Supplementary Figure S12. A maximum likelihood tree of MADS-box homologs of seven angiosperm species. The bootstrap values with 100 replicates are given for each node on the tree.

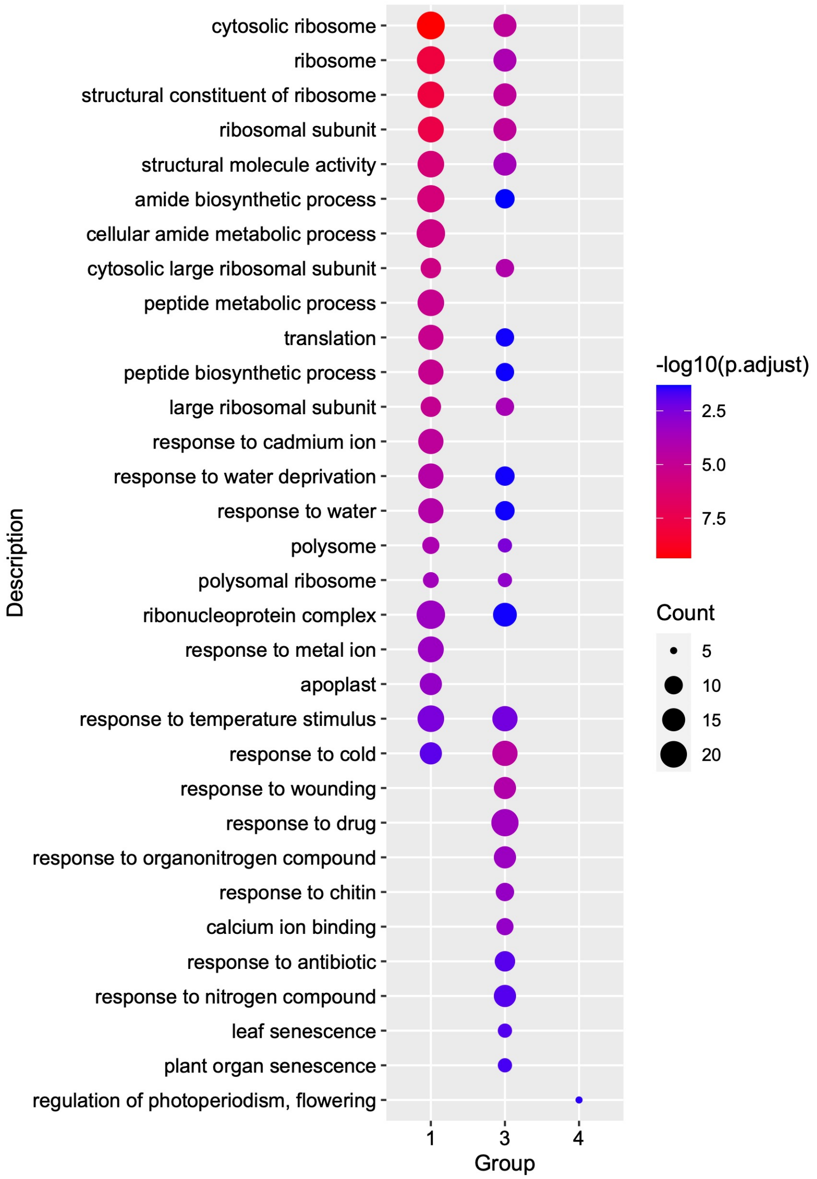

Supplementary Figure S13. Gene Ontology enrichment for the four clusters of genes in Figure 4C. Top 20 most enriched GO terms for each cluster are presented. Please note that no GO term is enriched for genes in cluster 2.

(The following figures are provided as a separate file)

Supplementary Figure S14. A maximum likelihood tree of auxin response factor (ARF) gene family of four angiosperm species. The bootstrap values with 100 replicates are given for each node on the tree. The size of dot on each node denotes for bootstrap values. *Phalaenopsis aphrodite*, *Oryza sativa*, *Arabidopsis thaliana* and *Amborella trichopoda*, are represented by Phap, Ory, Ara and Amb, respectively.

Supplementary Figure S15. A maximum likelihood tree of AUX/IAA gene family of four angiosperm species. The bootstrap values with 100 replicates are given for each node on the tree.

Supplementary Figure S16. A maximum likelihood tree of auxin inducible protein gene family of four angiosperm species. The bootstrap values with 100 replicates are given for each node on the tree.

Supplementary Figure S17. A maximum likelihood tree of cytokinin oxidase gene family of four angiosperm species. The bootstrap values with 100 replicates are given for each node on the tree.

Supplementary Figure S18. A maximum likelihood tree of cytokinin-responsive gata factor gene family of four angiosperm species. The bootstrap values with 100 replicates are given for each node on the tree.

Supplementary Figure S19. A maximum likelihood tree of bHLH gene family of four angiosperm species. The bootstrap values with 100 replicates are given for each node on the tree.

Supplementary Figure S20. A maximum likelihood tree of MYB gene family of four angiosperm species. The bootstrap values with 100 replicates are given for each node on the tree.

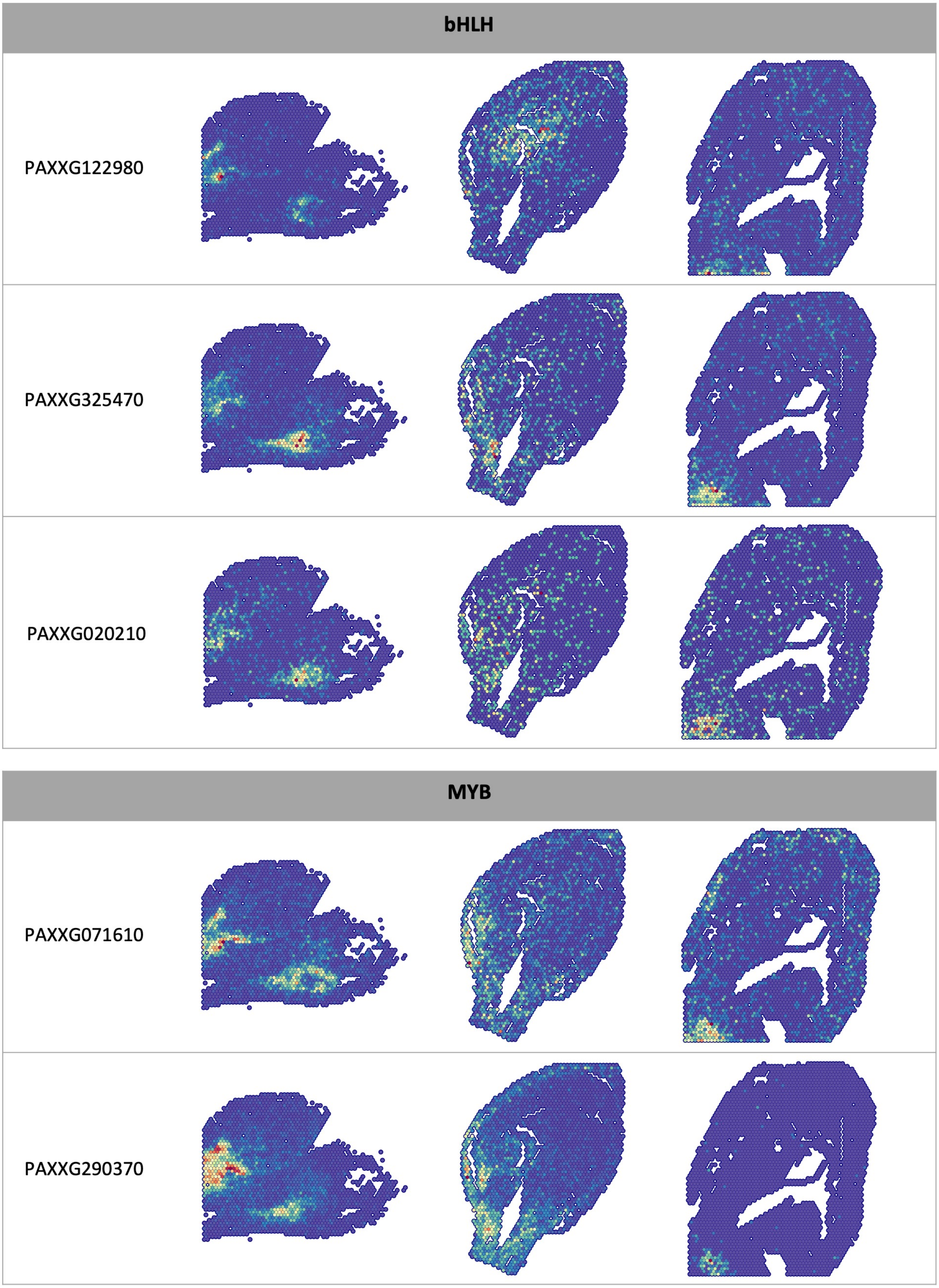

Supplementary Figure S21. Illustration of spatial expression of selected genes of bHLH and MYB gene families on different flowering stages.

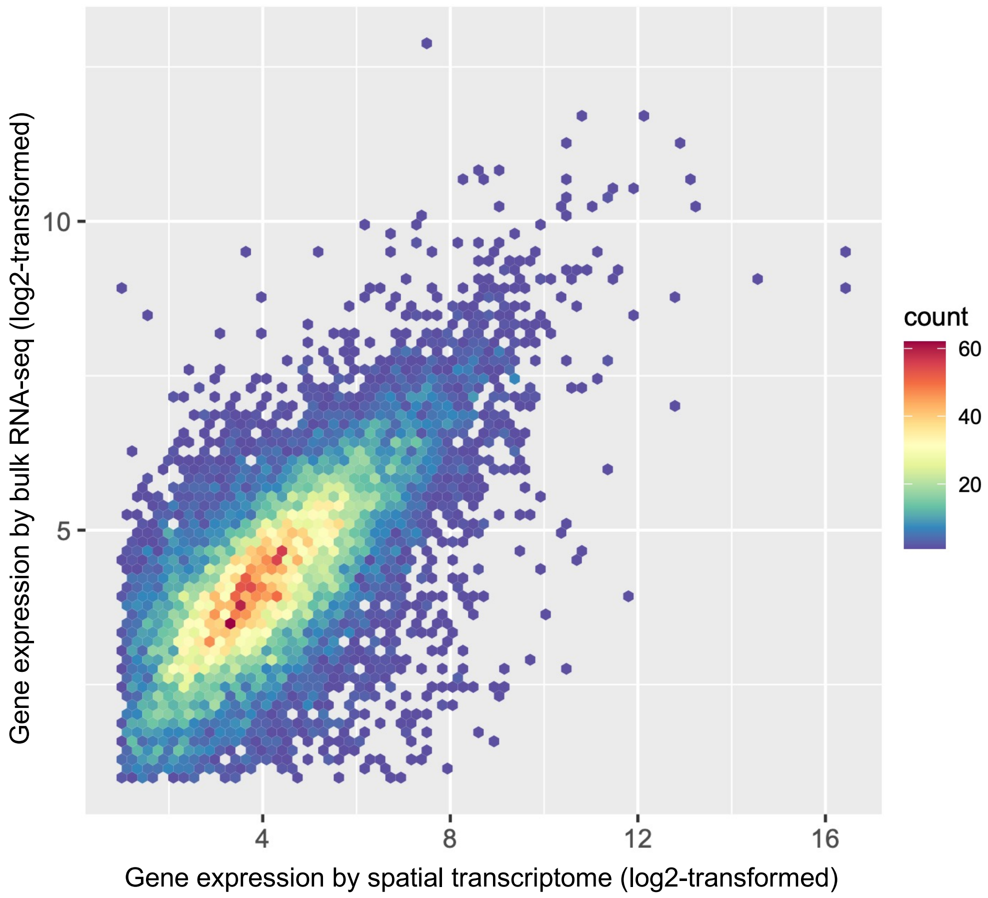

Supplementary Figure S22. Comparison of gene expression values of small flower bud detected by *P. aphrodite* by bulk RNA-seq (Chao et al. 2017, Plant Cell Physiology, NCBI GEO: SRR4302012) and by spatial transcriptome sequencing (bud 8). The FPKM values of the 13,090 genes shared by the two datasets show high consistency with Pearson's correlation coefficient as 0.68.
